# Lysine–arginine advanced glycation end‐product cross‐links and the effect on collagen structure: A molecular dynamics study

**DOI:** 10.1002/prot.26036

**Published:** 2020-12-23

**Authors:** Anthony Nash, Sang Young Noh, Helen L. Birch, Nora H. de Leeuw

**Affiliations:** ^1^ Nuffield Department of Clinical Neurosciences University of Oxford Oxford UK; ^2^ Department of Chemistry University of Warwick Coventry UK; ^3^ Department of Orthopaedics and Musculoskeletal Science Stanmore Campus, University College London London UK; ^4^ School of Chemistry University of Leeds Leeds UK

**Keywords:** advanced glycation end‐products, aging, collagen, cross‐linking, DOGDIC, glucosepane, matrix biology

## Abstract

The accumulation of advanced glycation end‐products is a fundamental process that is central to age‐related decline in musculoskeletal tissues and locomotor system function and other collagen‐rich tissues. However, although computational studies of advanced glycation end‐product cross‐links could be immensely valuable, this area remains largely unexplored given the limited availability of structural parameters for the derivation of force fields for Molecular Dynamics simulations. In this article, we present the bonded force constants, atomic partial charges and geometry of the arginine‐lysine cross‐links DOGDIC, GODIC, and MODIC. We have performed in vacuo Molecular Dynamics simulations to validate their implementation against quantum mechanical frequency calculations. A DOGDIC advanced glycation end‐product cross‐link was then inserted into a model collagen fibril to explore structural changes of collagen and dynamics in interstitial water. Unlike our previous studies of glucosepane, our findings suggest that intra‐collagen DOGDIC cross‐links furthers intra‐collagen peptide hydrogen‐bonding and does not promote the diffusion of water through the collagen triple helices.

## 1 INTRODUCTION

Collagen makes up approximately one‐third of protein in the human body. Skeletal tissues such as tendons, ligaments, cartilage and bone,^1^ the basement membrane,^2^ and the cornea^3^ are particularly rich in collagen. The mechanical properties of collagen are derived from its microscopic structure,^4^, ^5^ for example, type I collagen molecules assemble into fibrillar structures to lend structure and stability to the extracellular matrix (ECM). Within healthy young collagenous fibrils, enzyme‐mediated cross‐links form between the non‐helical telopeptide regions of neighboring collagen molecules, adding tensile strength and contributing to the desired bundling of collagen molecules.^6^ Owing to the extraordinarily long half‐life of some collagen types, up to 200 years in equine tendon^7^ and over 100 years in articular cartilage,^8^ adventitious chemical modifications, such as glycation events,^9^ result in the accumulation of further cross‐links by a spontaneous non‐enzymatic glycation reaction known as the Maillard reaction.^10^ In proteins, this reaction yields a post‐translational protein‐adduct between the dicarbonyl moieties of amino acid side chains within or between protein molecules,^11^ known as an advanced glycation end‐ product (AGE).

Studies have suggested that the accumulation of AGEs compromise tissue function, increase the susceptibility to injury and reduce healing capacity. In addition, AGE cross‐links are thought to contribute to several human pathologies, including neurological disorders,^12^ diabetes and cardiovascular disease,^13^ and rheumatoid arthritis.^14^ The AGE cross‐link glucosepane is known to be a major protein cross‐link of the senescent human matrix,^15^ accounting for >120 mol% of triple helical collagen modification in diabetic patients.^16^

In recent years, our group has simulated all‐atom models of fully solvated full‐length type I collagen,^17^, ^18^ with each model in the order of approximately 3000 amino acids in size. Our models have revealed sites that favor the formation of glucosepane.^17^ More recently, a study by Nash et al reported that an intra‐collagen glucosepane crosslink between two of the three collagen peptides making up the collagen protein, resulted in voids within the helical collagen protein.^15^ The inclusion of the bulky AGE allowed water to diffuse between collagen peptides, a finding which was supported by experimental evidence using differential scanning calorimetry. Unlike glucosepane, the 3‐deoxyglucosone‐ derived imidazolium cross‐link DOGDIC has not been shown to accumulate with age,^16^ which is likely to be the result of competition of crosslinking sites on the collagen molecules.^19^ Nevertheless, elevated levels of DOGDIC cross‐links have been found in patients with diabetes.^16^

To study AGEs at an atomistic level we require the complete derivation of bonded parameters of the arginine‐lysine methylglyoxal‐, glyoxal‐, and 3‐deoxyglucosone‐derived imidazolium cross‐links (MODIC, GODIC, and DOGDIC, respectively). Using ForceGen,^20^ a tool released by our group, we present a set of covalent bond length and covalent bond angle force constants with corresponding geometry values for DOGDIC, GODIC and MODIC cross‐links. A normal mode analysis was performed to analyze the geometry against quantum mechanical frequency analysis calculations. The parameters and atomic coordinates for DOGDIC were added into the same model collagen crystal structure as used in our earlier glucosepane studies. We then used Molecular Dynamics (MD) to investigate structural changes to cross‐linked collagen peptides and the dynamics of interstitial water. All force field and atom coordinate files have been made available to download (see Supporting Information for a list of simulation and data files).

## 2 COMPUTATIONAL METHODS

### 2.1 Electronic structure calculations

**FIGURE 1:**
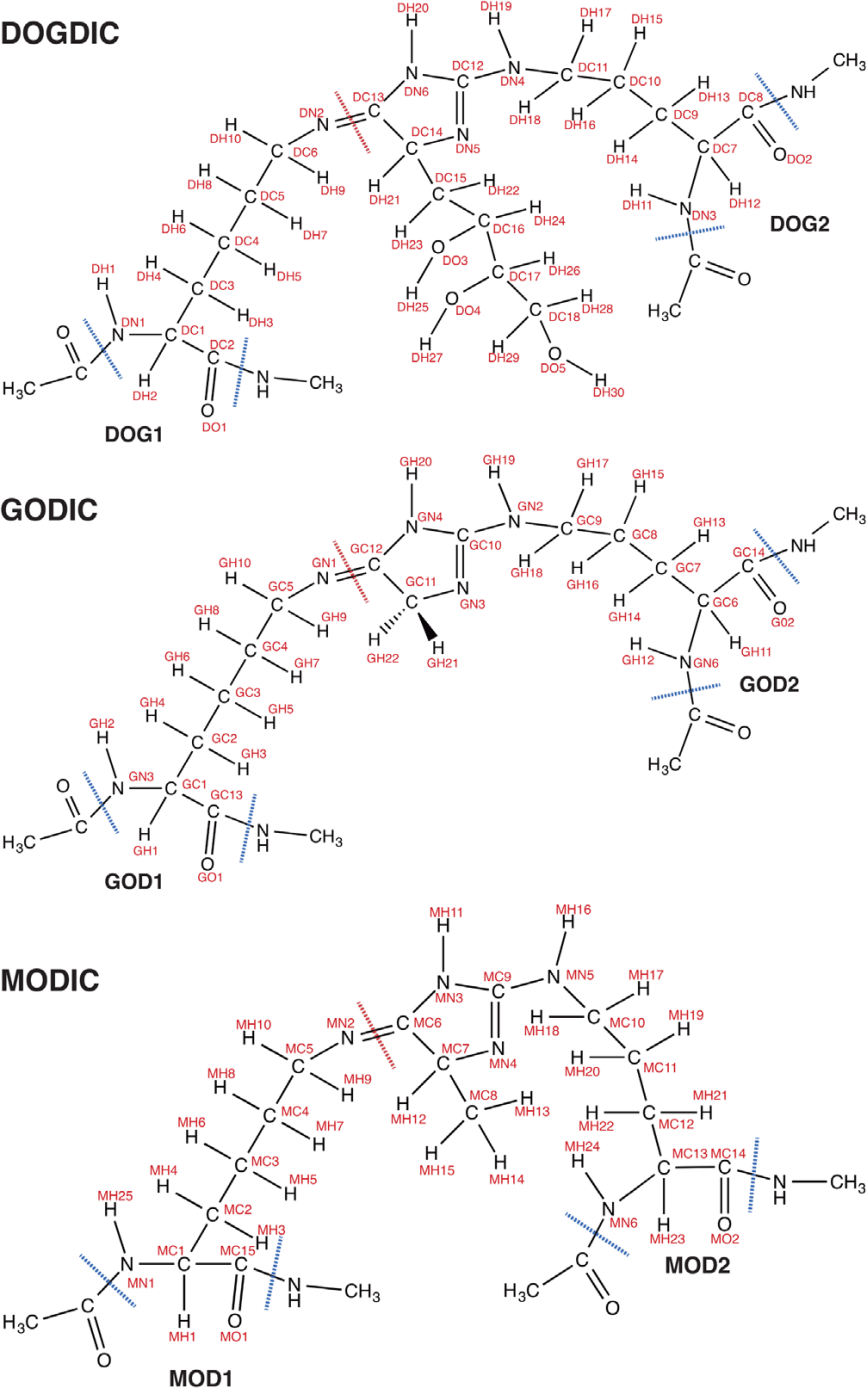
Atom labels used in the automated processing of force constants and geometry values within ForceGen. Peptide backbones are capped with methyl groups for the assignment of atomic partial charges (blue line), and the red line indicates a separation of one cross‐link into two distinct residues for its inclusion into Gromacs topology file [Color figure can be viewed at wileyonlinelibrary.com]

The structure of each AGE (Figure 1), was constructed with methyl‐capped backbone groups using Avogadro.^21^ A short steepest descent energy minimization procedure was performed to adjust inaccurate bond lengths and bond angles; at this stage, atom types were assigned using the Universal Force Field, pre‐packaged with Avogadro. Electronic structure optimization and frequency calculations were performed using Gaussian G09.^22^ The Hartree–Fock (HF) method and a 6‐31G(p) basis set was employed, according to the Amber forcefield,^23^ for electronic structure optimization with a tight threshold, that is, 1.5 ×10^5^, 1 ×10^5^, 6 ×10^5^, 4 × 10^5^, for maximum force, root mean squared (RMS) force, maximum displacement, and RMS displacement, respectively. Optimized structures were checked for imaginary frequencies using vibrational frequency analysis. *Ab inito* calculations were performed in a vacuum.

### 2.2 Bonded force constant derivation

In this study, we approached the derivation of atomistic bonded force constants through the diagonalisation of the Hessian. The Hessian originates from the vibrational frequencies of an optimized cross‐link and describes the second order of the potential energy of the molecular system with respect to a small change in atomistic position.^24^ We used ForceGen to yield bond length and bond angle force constants and geometry values. A unique set of atom types and atom names were defined and used to populate two input files, one for the desired bonded atom pairs and the other for the bond angle atom triplets. The atom types were accompanied by the matching atom ID from the Gaussian output files. Further instructions can be found at the software website: https://sourceforge.net/projects/forcegen/ and https://github.com/acnash/UCLCollagenGroup.

### 2.3 Restrained electrostatic partial charge derivation

The HF/6‐31G(p) optimized structures were submitted to the pyRED server (through an on‐line submission to the R.E.D [RESP ESP charge Derive] Server Development).^25^ Gaussian geometry optimization was switched off and the capped groups were restrained for RESP charge fitting to the central fragment resulting in a zero‐net charge. Partial atomic charges compatible with the Amber99SB‐ildn force field were assigned to each AGE structure via an entry in the Gromacs aminoacid.rtp force field file.

### 2.4 Model construction

The model building steps were replicated using the methods of Nash et al.^15^ A short description is given here, and an accompanying guide is linked in the Supporting Information. Two models were constructed; a collagen wildtype (WT model), and the collagen model cross‐linked with a DOGDIC AGE (AGE model). The collagen content in both models was duplicated across two dimensions to increase the system size and prevent periodic boundary condition artifacts.

**FIGURE 2:**
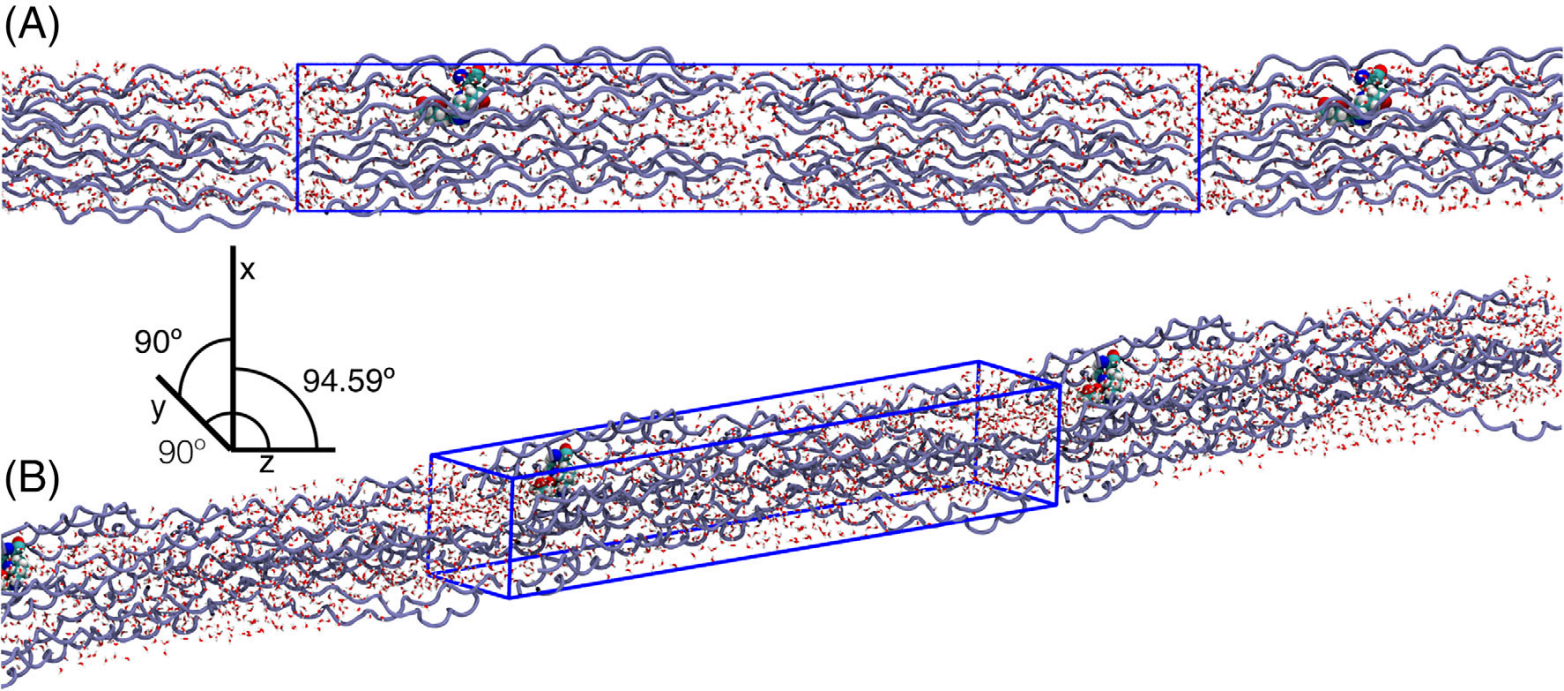
A representation of the constructed MD model from a side profile A, and a tilted orientation B, to illustrate the unit cell shape. Collagen peptides are presented in blue, water in red, and the location of the DOGDIC cross‐link in spheres. The unit cell has been replicated to give a perspective of the fibrillar‐like nature of the model [Color figure can be viewed at wileyonlinelibrary.com]

The WT model was constructed using the 4Z1R^26^ crystal structure with an asymmetric unit cell. All water was removed, and incomplete sidechains were recovered using the Protein Preparation Wizard in Schrödinger Maestro. An additional cell was constructed by replicating the asymmetric unit cell on the y‐axis. To prevent the short‐range interaction distance from being longer than half the unit cell dimension, the four molecules and the crystallographic dimensions were replicated normal to the long axis, yielding a total of eight collagen proteins (Figure 2). These adjustments were made using the CCP4 software package.^27^ The unit cell was solvated using tip3p water molecules from the Amber99SB‐ildn force field. The system had net zero charge.

The AGE model was constructed by replicating the final unit cell of the WT model (with water removed) and then removing two amino acids (including the corresponding peptide backbone) on the same collagen protein but different polypeptide chains. The DOGDIC structure was built using the Build Tool in Avogadro and then positioned by optimizing the distance between the peptide bonds of the existing backbone with the backbone of the cross‐linked amino acids whilst keeping the unaltered collagen fixed. The force constants for bond length and bond angle, atom names and types, atomic partial charges, and non‐bonded interaction forces were added to the GROMACS Amber99SB‐ildn forcefield files, having been parameterized using the electronic structure calculations and bond force constant derivation steps documented above. Dihedral angle force constants were taken from existing force field values that best matched the structure of the cross‐link.

### 2.5 Molecular dynamics simulation details

Molecular Dynamics simulations were performed using Gromacs version 2018.2^28^ with the leap‐frog integrator and a time‐step of 0.002 ps. Hydrogen bonds were holonomically constrained using the LINCS constraint algorithm with a LINCS iteration of 1 and LINCS order of 4. Neighborhood list updates were calculated using the Verlet cut‐off scheme and updated every 10 steps (20 fs). Electrostatic charges were treated using the Particle Mesh Ewald (PME) scheme with a PME order of 4 and modified using a potential‐shift. Temperature fluctuated about the mean of 312.1 K ± 3.4 K and was controlled using velocity rescaling with a time constant of 0.1 ps. Pressure was set to 1 bar and controlled using uniform scaling of the unit cell with the Parrinello–Rahman scheme and updated every 2 ps. A 1 nm short‐range LJ‐6‐12 cut‐off was applied and modified using a potential‐shift. Energies, velocities, and coordinates were collected every 20 ps.

### 2.6 System equilibration

**FIGURE 3:**
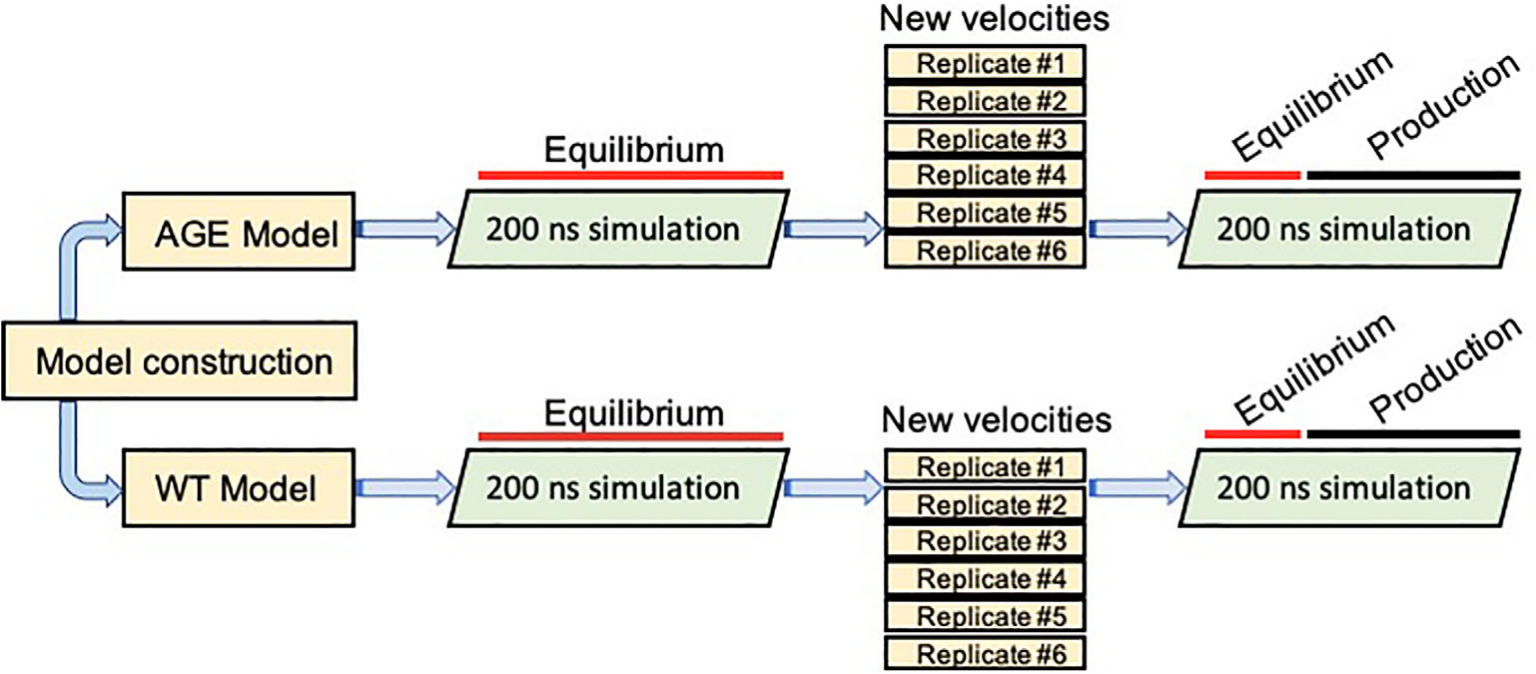
An overview of the study design from construction of both models through to replicate simulations. The red and black bars represent the proportion of recorded frames used for equilibration of the system and production analysis, respectively [Color figure can be viewed at wileyonlinelibrary.com]

Both WT and AGE systems were relaxed using the following steps (see Figure 3 for an overview of the study design). Firstly, the solvated systems were subjected to an energy minimization using a steepest gradient descent. The systems were left to adjust until all forces had dropped below 1000 kJ mol^−1^. A 1000 kJ mol^−1^ position restraint (across three dimensions) was applied to the collagen backbone heavy atoms. Both energy‐minimized structures were subjected to a 50 ps NVT (fixed particle, volume, and temperature) simulation using the Berendsen thermostat coupling. The velocities were preserved, the temperature coupling was replaced with the velocity rescaling scheme and a Berendsen pressure‐coupling was applied. Both restrained systems adjusted for a further 50 ps using the NPT ensemble (fixed particles, pressure, and temperature). Finally, position restraints were removed, the pressure coupling was replaced with the Parrinello–Rahman scheme and both systems were left to run for 200 ns to equilibrate.

The final frame coordinate data for the WT model and AGE model were then used to start six simulations per model using new velocities. The simulations were left to run for 200 ns of which the final 150 ns were used for production run analyses. Production run data (Gromacs tpr, cpt, and mdp files) are available via the Supporting Information.

## 3 RESULTS AND DISCUSSION

The first half of this study reports the calculated force field parameters for the three AGE cross‐links, which were then analyzed by comparing MD normal mode analysis to QM (quantum mechanical) calculations. The second half of this study features an investigation on the presence of a DOGDIC cross‐link within a solvated all‐atom collagen model.

### 3.1 Force constant derivation

Cross‐links DOGDIC, GODIC and MODIC were constructed using Avogadro described above to match the corresponding schematic in Figure 1. Methyl groups were used to terminate each backbone and the system could reach a crude energy minimum. Structures were then resolved further using ab initio calculations as described. Final frequency calculations were performed over the optimized geometry to check for imaginary frequencies and to yield the Hessian.

To calculate restrained atomic partial charges the Gaussian output text file from each QM structure calculation was up‐loaded to pyRED. Each cross‐link was composed from two residue entries in the aminoacids.rtp force field file (a complete net partial charge of zero) and combined with an entry in the Gromacs topology specbond.dat file. A Gaussian ASCII output text file and a corresponding formatted checkpoint file were loaded into ForceGen for the complete description of all bond length and bond angle force constants and geometry values. The atomic partial charges and bond length values for DOGDIC are presented in Table S1, for GODIC in Table S3, and for MODIC in Table S5. Bond angle force constants and bond angle values for DOGDIC are presented in Table S2, for GODIC in Table S4, and for MODIC in Table S6. The bond lengths necessary to connect each cross‐link half between two peptide chains in Gromacs is presented in Table S7.

### 3.2 Structural analysis of derived force constants

**FIGURE 4:**
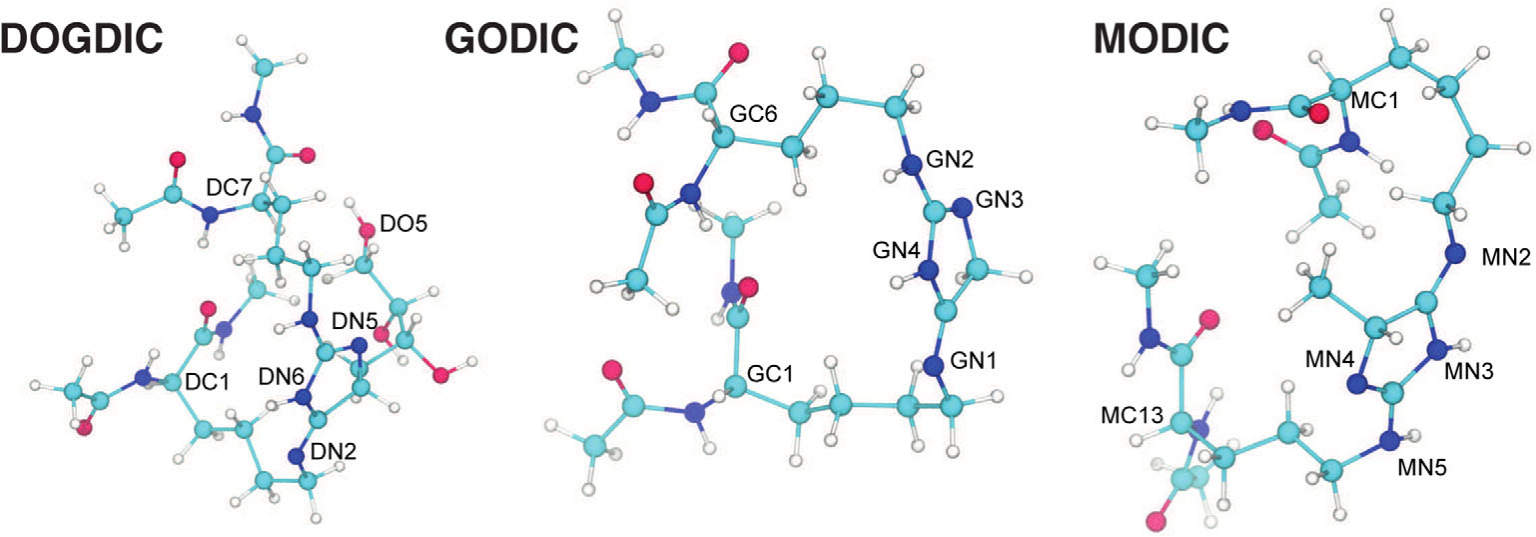
Atomic structures of the three arginine‐lysine cross‐links DOGDIC, GODIC, and MODIC, taken from the energy minimum of electronic structure calculations. Each cross‐link includes a neutralized peptide backbone. Key atoms have been labeled to help orientate the reader. Elements are represented by colored spheres: hydrogen ‐ white, nitrogen ‐ blue, oxygen ‐ red, and carbon ‐ teal. Key atoms have been labeled to help orientate the reader [Color figure can be viewed at wileyonlinelibrary.com]

Structural stability from the derived atomic partial charges and the force constants for bond length and bond angles of each cross‐link was analyzed using short cross‐linked peptides. The N‐ and C‐terminal backbones of each minimum energy cross‐link structure (Figure 4) were terminated with neutral‐backbone capped glycine residues. A unit‐cell of 4.0 nm × 4.0 nm × 4.0 nm was used, which is large enough to avoid periodic boundary interaction artifacts. The system was constructed in a vacuum to replicate the above ab initio optimization set‐up. Each cross‐link system was minimized using an iterative combination of conjugate gradients and L‐BFGS energy minimization techniques in double precision until a maximum force threshold of 0.001 kJ mol^1^ or less was reached.

**FIGURE 5:**
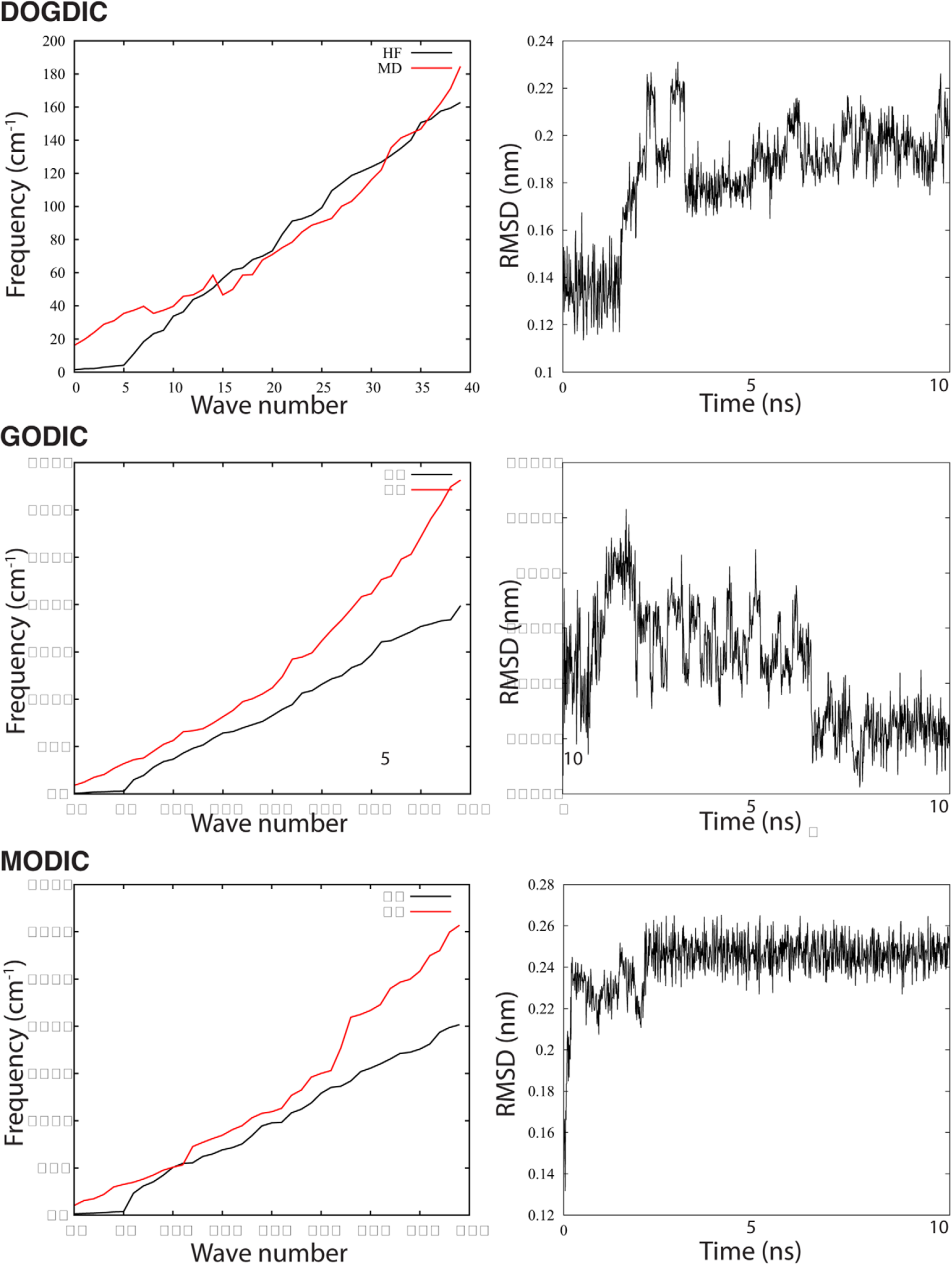
The molecular dynamics derived normal‐mode analysis over the first 40 wave numbers compared with vibrational frequency analysis from ab initio optimized structures and accompanied by the root mean squared deviation over a 10 ns simulation [Color figure can be viewed at wileyonlinelibrary.com]

A set of molecular eigenvalues was derived from normal mode analysis using Gromacs. These were compared to the vibrational frequencies from the HF electronic structure calculations used to derive the force constant. As seen in Figure 5, the first six wave numbers, that is, the three translational and three rotational degrees of freedom for the whole molecule, are very small. Then, there is a similar trend in frequency with a growing deviation as the wave number increases, which is indicative of the difficulty in reproducing frequency modes in peptides. It is also likely that different energy minima will produce different frequencies, and, unlike the ab initio calculations, the normal‐mode analysis will be affected by the four glycine residues. Finally, each cross‐linked system was subjected to a 100 ps simulation using the velocity rescaling thermostat at 298 K, before switching to a Nosé–Hoover thermostat with a pressure‐coupling set to one atmosphere and left to evolve for 10 ns. As seen in Figure 5, for a molecular model using paired potentials, the root‐mean‐square deviation (RMSD) falls within expected thermal fluctuation levels, demonstrating a stable structure. It should be noted that the addition of the four glycine residues will affect the RMSD time‐series and we expect any significant deviation from the resolved electronic structure of the AGE to be partial effected by these neighboring residues.

### 3.3 Molecular dynamics of arginine‐lysine crosslink collagen

#### 3.3.1 Conformational sampling

Production simulations were performed for 200 ns using the NPT ensemble, as described in the methods section. For each system, the AGE model (DOGDIC cross‐linked) and the WT model (pure collagen without an AGE crosslink), were simulated in six replicates using randomly assigned velocities for the first step from a Boltzmann distribution at 312 K. Multiple simulations with randomly assigned velocities were used to improve sampling of conformational space and improve statistical quality in the analysis.^29^ The temperature distribution (Figure S2) had a mean of 312 K, the pressure remained consistent (Figure S3) along with the unit cell volume (Figure S4), and the potential energy distributions between each replicate were consistent (Figure S1).

The RMSD of the backbone heavy atoms from each collagen molecule for all replicates (Figure S5) revealed little change after the initial 50 ns. However, two of the WT model replicates demonstrated subtle changes (0.5‐1 Å compared to the other four replicates) after 150 ns. The AGE model replicates were highly consistent throughout the production run simulations. A set of net RMSD calculations (ones in which the RMSD of the backbone heavy atoms are calculated against every previous frame) revealed several micro‐states within each replicate trajectory (Figure S6 and S7) where in particular the trajectory of the first and third WT model replicates revealed different conformational states that correspond well with the basic RMSD calculation presented earlier. In contrast, the net RMSD calculations for the AGE model replicates show only very subtle changes in conformational states, which is consistent with the earlier RMSD backbone calculations. We allowed each simulation to settle for the first 50 ns and only the 50 to 200 ns timespan was used for further structural and water analysis.

We explored the variation in conformational sampling by calculating the variance in backbone motion over the production run using principal component analysis (PCA). PCA is an exploratory data analysis technique often used to visualize data co‐variances across vectors called principal components (PC). The initial set of PCs capture the greatest variance in a system. In Molecular Dynamics simulations variances plateau after approximately 15 to 20 PCs with the first set of PCs representing the greatest amount of conformational variance. PCA of all six WT and AGE model replicates (Figures S8 and S9) demonstrates at least two structural minima in all but one case (replicate 3 of AGE model). There was no redundancy in conformational sampling amongst replicates, suggesting extensive conformational sampling.

#### 3.3.2 Collagen structure and interstitial water analysis

The ECM comprises several hydrophilic components that either bind to or are near collagen. For example, sulphated and non‐sulphated glycosaminoglycan (GAG) chains provide water retention properties to the ECM.^30^ Both GAGs provide hydration and water transport to the human dermis.^31^, ^32^ We reported in earlier glucosepane studies that aged tissue, with increased levels of glucosepane cross‐links, retained more water.^15^ Whilst preparing this manuscript, experimental information on DOGDIC intra‐ and inter‐molecular hydrogen bonding was not available, however, one would expect some similarity to Glucosepane.

The hydrogen‐bond capacity of DOGDIC with the water environment and between the three collagen chains making up a collagen molecule were calculated using MDAnalysis.^33^ Hydrogen‐bonds were defined using typical protein bond acceptor and donor atoms with a bond distance of 3 Å and bond angle criteria of 120°. Data points were obtained between 50 ns and 200 ns and all six replicates were combined. DOGDIC donor atoms were: HD10, HD12, HD13, HD5, HD6, HN, and the acceptor atoms were: N, ND2, ND4, ND5, ND6, OD1, OD2, OD3, OD4, OD5 (Figure 1). All hydrogen‐bond analyses were performed for all six replicates and the data output was combined. Where a comparison between distributions was required, each distribution was first tested for normality, and then based on that result an appropriate test for the difference between distributions was chosen.

**FIGURE 6:**
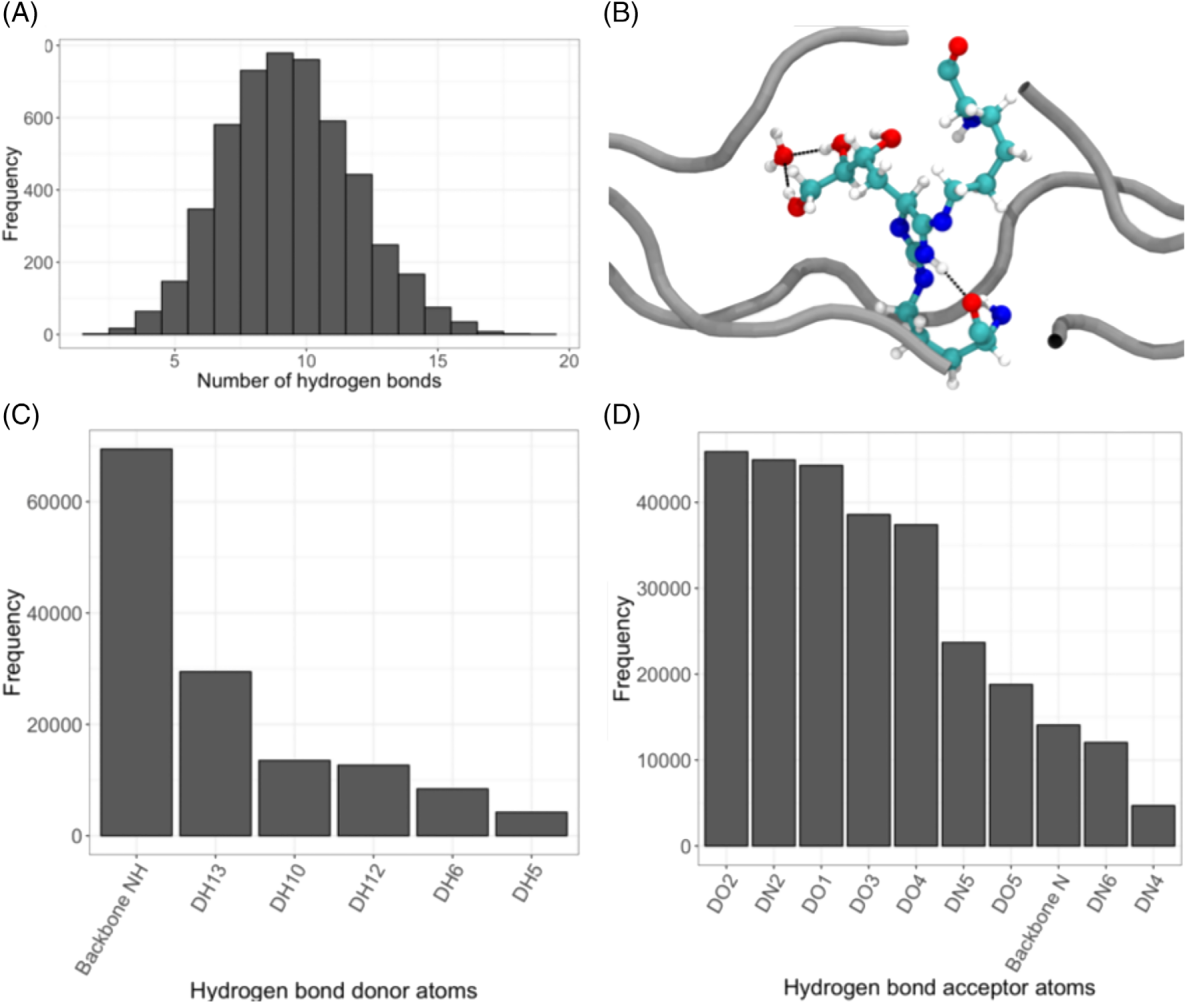
The result of hydrogen bond analysis between the DOGDIC AGE cross‐link and the water environment. A, The number of hydrogen‐bonds take on an almost normal distribution. B, A single frame representation of hydrogen bonding between water and the polar hydroxyl groups of the cross‐link. C, the number of recorded hydrogen bonds by bond donor and D, by bond acceptor. The atom naming is consistent with those presented in the DOGDIC cross‐link schematic [Color figure can be viewed at wileyonlinelibrary.com]

The hydrogen‐bond distribution between DOGDIC and water was calculated (Figure 6A), resulting in a median of nine bonds, a minimum of two bonds and a maximum of 19 bonds. The average number of bonds was 9.4 with a SD of 2.5. The frequency of atom‐bond participation revealed that, despite the many number of polar regions on the cross‐link, the backbone nitrogen was predominately involved as a donor (Figure 6C), whilst the hydroxyl groups along the cross‐link (Figure 6D) were predominately involved in hydrogen‐bond formation, more so than the backbone nitrogen or nitrogen atoms proximal to the crosslink imidazole ring. A representation of the intra‐collagen cross‐link interacting with water is presented in Figure 6B.

**FIGURE 7:**
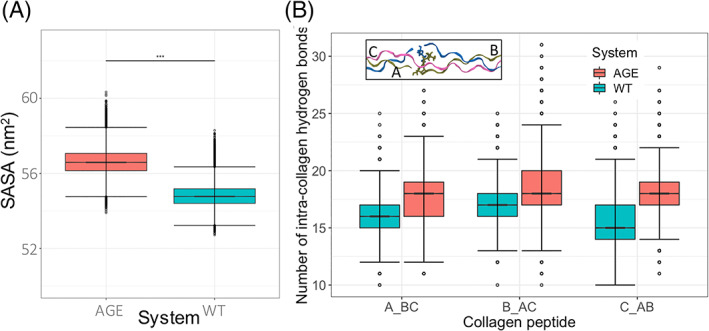
A, The combined solvent accessible surface area calculations of all six replicates of the AGE model and WT Model presented as a box‐whisker distribution. The median, interquartile range and statistical significance between are presented. B, The distribution of hydrogen‐bonds between a collagen polypeptide chain and the two other collagen polypeptide chains (labeled A‐C in the inset). In the WT model, a single collagen protein was selected, in the AGE model, the collagen with the cross‐link was selected [Color figure can be viewed at wileyonlinelibrary.com]

**FIGURE 8:**
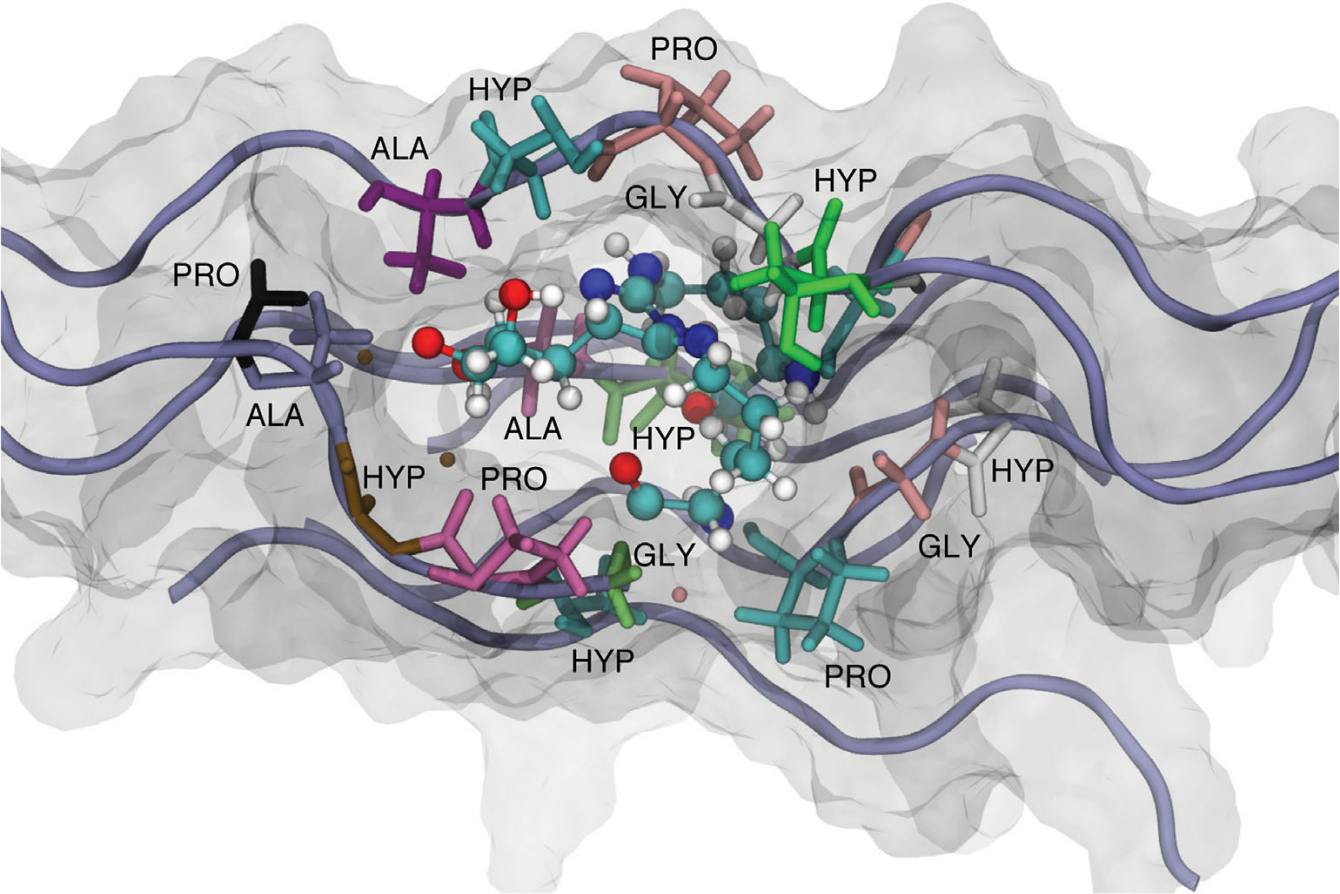
A visualization of the centroid structure from the combined trajectories of the AGE model. The structure was isolated having first calculated the RMSD of all pairwise conformations and then transformed these distances into similarity scores. The structure with the maximum similarity score across the combined trajectories was visualized. The collagen is represented in blue tube, semi‐transparent Van der Waals space filling and the immediate neighboring amino acids are presented and labeled accordingly. The DOGDIC crosslink can be seen aligned parallel with the collagen polypeptide chains [Color figure can be viewed at wileyonlinelibrary.com]

We have calculated the solvent accessible surface area (SASA) trajectory distribution of a single collagen molecule in the WT model and the cross‐linked collagen molecule in the AGE model. We used Shrake and Rupley's implementation of SASA as presented in the MDTraj software suit.^34^ The default parameters of 960 points per atom and an atomic radius of 0.14 nm was used. A test of normality was performed using the Shapiro‐Wilk test (*P* < .001) and a test of significance difference in the median (Fligner–Killeen) suggested that the cross‐linked AGE model was more accessible to solvent than the WT model (Figure 7A). However, although the differences were significant, in relative terms these differences are not huge. The addition of the large cross‐link (Figure 8) may be responsible for this subtle increase in surface area.

Collagen maintains a triple helix structure via a network of intra‐collagen hydrogen‐bonds between the backbone nitrogen and oxygen donor and acceptor atoms.^35^ We have calculated the distribution of intra‐collagen hydrogen‐bonds between the WT model and the AGE model across all replicates (Figure 7B). In all three instances of a polypeptide chain interacting with its two immediate neighbors (chains A…BC, B…AC, and C…AB), the WT model has consistently fewer hydrogen‐bonds than the AGE model. A non‐normal distribution was confirmed using the Shapiro‐Wilk test (*P* < .001) and a Fligner–Killeen test of pair‐wise comparison of variance revealed a significant difference in median number of hydrogen‐bonds between AGE and WT models. The presence of an AGE crosslink increased the median number of hydrogen‐bonds from 16, 17, and 15 to 18 in the intra‐collagen peptides A…BC, B…AC, and C…AB, respectively.

The increased number of hydrogen‐bonds in the AGE model agrees with the position of the cross‐link relative to the collagen polypeptide chains. Unlike glucosepane with its larger seven membered ring, DOGDIC has a much smaller imidazole ring. This suggests that as an intra‐collagen cross‐link, DOGDIC can pack parallel to the collagen backbone as observed in these simulations (Figure 8). The packing would result in an increased solvent accessible surface area, as observed, and an increase in intra‐collagen hydrogen‐bonds, whilst still being able to bond with interstitial water, which was also observed.

## 4 CONCLUSION

Reports from The Office for National Statistics (UK) show that there are more people over 65 than children under 16. It is further expected that in 20 years' time over half of the UK adult population will be over 50 and predictions suggest that children born today are expected to live beyond 100. We are fortunate to see such long‐life spans when half a century ago only one in 10 children would live into their 90s. However, this longevity comes at the cost of managing the endemic of diseases associated with an aging population. It is believed that the accumulation in AGEs is a fundamental process central to age‐related decline in musculoskeletal tissues and locomotor system function. AGE cross‐link formation, specifically arginine‐lysine cross‐links, within the tendon collagenous matrix is thought to render it more resistant to proteolytic degradation, thereby allowing matrix damage to accumulate and mechanical properties to deteriorate. However, studies at the atomistic level of collagen molecular packing are few. This article presents the bonded parameters for DOGDIC, GODIC and MODIC to enable further study by those in this field of computational chemistry.

Our implementation of an intra‐collagen DOGDIC cross‐link into a model collagen presents different behavior from that uncovered in our earlier studies on glucosepane. Whereas glucosepane was shown to increase the interstitial water presence, our DOGDIC simulations reveal a cross‐link that does not disturb the local collagen structure; in fact, the many polar regions and the small imidazole‐like ring could contribute to intra‐collagen polypeptide packing.

There are limitations worth noting. Firstly, although the parameterization of the AGE‐cross links was calculated using an established ab initio method, there is currently, to the author's knowledge, no known structurally resolved data to validate the force field parameters. This limitation should be taken into consideration when interpreting the results. Secondly, the AGE cross‐link was positioned within the intra‐collagen space rather than between collagen molecules. Although there has been significant effort in determining glycation reaction sites,^15^ it is still unknown whether in vivo formation favors intra‐ or inter‐collagen locations. Finally, the collagen crystal structures presented are significantly shorter than a complete type I collagen protein, which measures just short of 300 nm. However, the cross‐linking of shorter model collagen fragments has yielded results that compare well with experimental data of human tendon tissue.^15^

### PEER REVIEW

The peer review history for this article is available at https://publons.com/publon/10.1002/prot.26036.

## Supporting information


**Appendix** **S1.** Supporting Information.
