# Supplementary material for: Lysine–arginine advanced glycation end‐product cross‐links and the effect on collagen structure: A molecular dynamics study
Source: Proteins. 2020 Dec 23;89(5):521–30. doi: 10.1002/prot.26036 (PMC8048459; doi:10.1002/prot.26036)
Supplement: Supplementary file 1 — Appendix S1. Supporting Information. [file PROT-89-521-s001.pdf]

Supplementary Information: Lysine-Arginine Advanced Glycation End-Product Cross-links and the Effect on Collagen Structure: A Molecular Dynamics Study.

Anthony Nash[1]*, Sang Young Noh[2], Helen L. Birch[3], Nora H. de Leeuw[4]

[1]Nuffield Department of Clinical Neurosciences, University of Oxford, Oxford, UK, OX3 9DU (https://orcid.org/0000-0001-8212-0302)

[2]Department of Chemistry, University of Warwick, Coventry, CV4 7EQ, UK

[3]Department of Orthopaedics and Musculoskeletal Science, Stanmore Campus, University College London, London, HA7 4LP, UK

[4]School of Chemistry, University of Leeds, Leeds, LS2 9JT, UK (https://orcid.org/0000-0002-8271-0545)

*Corresponding author: anthony.nash@ndcn.ox.ac.uk

Simulation data files

The following files can be downloaded from Github: https://github.com/acnash/AGES_DOGDIC
-DOGDIC AGE Gromacs .tpr files
-DOGDIC AGE Gromacs .cpt files
-DOGDIC AGE Gromacs .gro files
-Wildtype Gromacs .tpr files
-Wildtype Gromacs .cpt files
-Wildtype Gromacs .gro files
-Modified AMBER force field files for Gromacs
-Gromacs mdp run parameter files
-DOGDIC AGE Gromacs topology file (.itp)

-Wildtype Gromacs topology file (.itp)
-DOGDIC AGE system-wide Gromacs topology file (.itp)
-Wildtype system-wide Gromacs topology file (.itp)
-DOGDIC AGE Gromacs index file (.ndx)
-Wildtype Gromacs index file (.ndx)
-DOGDIC AGE PCA data files
-Wildtype PCA data files

Force constant derivation

SI Table 1: The complete set of restrained atomic partial charge values for DOGDIC along with atomic pair bond length force constants (FC) in kJ mol^{-1} and corresponding equilibrium lengths (nm).

Atom	Charge	Atom	Charge	Bond	Length	FC	Bond	Length	FC	Bond	Length	FC
DN1	-0.3394	DC10	0.0127	DN1 DH1	0.099	171548.261	DC9 DH14	0.109	127095.404	DC18 DH29	0.109	136564.454
DH1	0.2125	DH15	0.0329	DN1 DC1	0.144	171548.261	DC9 DC10	0.153	105841.202	DC18 DO5	0.139	69438.079
DC1	-0.0601	DH16	0.0329	DC1 DH2	0.108	153399.965	DC10 DH15	0.109	405220.497	DO5 DH30	0.095	96059.354
DH2	0.0916	DC11	0.0306	DC1 DC2	0.153	89908.146	DC10 DH16	0.108	138813.491			
DC2	0.5414	DH17	0.0837	DC2 DO1	0.122	315482.806	DC10 DC11	0.153	109300.079			
DO1	-0.4646	DH18	0.0837	DC1 DC3	0.154	84206.191	DC11 DH17	0.108	107670.503			
DC3	-0.0126	DN4	-0.7517	DC3 DH3	0.108	45856.944	DC11 DH18	0.108	67798.216			
DH3	0.0216	DH19	0.3830	DC3 DH4	0.109	44286.368	DC11 DN4	0.146	121228.221			
DH4	0.0216	DC12	0.7837	DC3 DC4	0.153	93448.692	DN4 DH19	0.100	221292.284			
DC4	0.0114	DN5	-0.6837	DC4 DH5	0.108	152748.149	DN4 DC12	0.137	160604.034			
DH5	0.0034	DN6	-0.6853	DC4 DH6	0.109	132493.413	DC12 DN5	0.126	266534.962			
DH6	0.0034	DH20	0.3616	DC4 DC5	0.153	95415.177	DC12 DN6	0.138	153142.711			
DC5	-0.0006	DC13	0.5005	DC5 DH7	0.109	89672.313	DN6 DH20	0.100	82736.086			
DH7	0.0190	DC14	0.2385	DC5 DH8	0.109	178049.914	DN6 DC13	0.140	99438.630			
DH8	0.0190	DH21	0.0570	DC5 DC6	0.154	105352.500	DC13 DC14	0.155	95552.445			
DC6	0.1004	DC15	-0.0161	DC6 DH9	0.108	110735.315	DC14 DN5	0.146	101817.935			
DH9	0.0323	DH22	0.0576	DC6 DH10	0.109	124359.421	DC14 DH21	0.109	138339.658			
DH10	0.0323	DH23	0.0576	DC6 DN2	0.144	63531.021	DC14 DC15	0.153	53330.327			

DN2	-0.6124	DC16	0.0435	N DC2	0.133	176436.798	DC15 DH22	0.108	149115.074			
DN3	0.4625	DH24	0.0764	C DN1	0.135	162246.033	DC15 DH23	0.108	150363.975			
DH11	0.2490	DO3	-0.7057	DN2 DC13	0.125	186307.095	DC15 DC16	0.153	106887.426			
DC7	-0.0118	DH25	0.4552	N DC8	0.133	176423.750	DC16 DH24	0.108	85574.655			
DH12	0.0889	DC17	0.1044	DC8 DO2	0.121	325547.562	DC16 DO3	0.142	140524.412			
DC8	0.6382	DH26	0.0965	DC8 DC7	0.153	96705.913	DO3 DH25	0.095	238922.320			
DO2	-0.5468	DO4	0.5962	DC7 DH12	0.108	115294.316	DC16 DC17	0.153	96996.331			
DC9	-0.0632	DH27	0.4162	DC7 DN3	0.146	116086.542	DC17 DH26	0.109	123848.916			
DH13	0.0303	DC18	0.1135	DN3 DH11	0.100	139423.472	DC17 DO4	0.140	87739.129			
DH14	0.0303	DH28	0.0353	DN3 C	0.134	190916.125	DO4 DH27	0.095	102129.168			
DH29	0.0353	DO5	-0.5938	DC7 DC9	0.154	84120.874	DC17 DC18	0.152	110262.387			
DH30	0.3676			DC9 DH13	0.108	115947.250	DC18 DH28	0.109	74839.712			

SI Table 2: The complete set of bond angle force constants (FC) in kJ mol^{-1} and corresponding equilibrium angle (degree) for DOGDIC.

Triplet	Angle	FC	Triplet	Angle	FC	Triplet	Angle	FC
C DN3 DC7	126.671	292.174	DH23 DC15 DC16	107.002	203.476	DC15 DC16 DH24	109.717	270.997
DC11 DN4 DH19	114.348	107.553	DH3 DC3 DC4	110.792	223.653	DC12 DN4 DH19	114.312	95.391
DH20 DN6 DC13	119.864	254.587	DH4 DC3 DC4	109.792	333.267	DC12 DN4 DC11	117.280	372.264
DH22 DC15 DC16	110.645	96.376	DC4 DC5 DC6	114.036	320.613	DC12 DN5 DC14	107.900	307.053
DH23 DC15 DC16	107.002	203.476	DC4 DC5 DH7	108.802	119.281	DN5 DC12 DN4	125.336	256.104
DH13 DC9 DC10	111.124	193.321	DC4 DC5 DH8	110.177	155.265	DN5 DC14 DH21	110.407	183.393
DH14 DC9 DC10	109.541	121.168	DH5 DC4 DH6	106.692	108.313	DN5 DC14 DC15	108.164	233.470
CT N DC2	124.326	278.510	DH5 DC4 DC5	108.398	250.275	DH21 DC14 DC15	109.249	206.802
H N DC2	117.820	262.167	DH6 DC4 DC5	109.848	144.524	DC14 DC15 DH22	107.988	116.422
N DC2 DC1	115.327	361.208	DH7 DC5 DH8	106.808	174.098	DC14 DC15 DH23	112.044	137.072
N DC2 DO1	123.562	356.408	DH7 DC5 DC6	108.563	253.470	DC14 DC15 DC16	111.704	300.983
DO1 DC2 DC1	121.111	347.062	DH8 DC5 DC6	108.199	194.943	DH23 DC15 DH22	107.391	99.005
DC2 DC1 DH2	108.461	113.542	DC5 DC6 DN2	110.549	297.334	DC15 DC16 DH24	109.717	270.997
DC2 DC1 DC3	112.632	263.616	DC5 DC6 DH9	110.206	185.801	DC15 DC16 DO3	105.875	187.802
DC2 DC1 DN1	107.701	271.467	DC5 DC6 DH10	108.103	245.366	DC15 DC16 DC17	113.533	303.231
DH2 DC1 DC3	107.952	108.826	DH9 DC6 DH10	106.519	137.421	DH24 DC16 DO3	127.928	19.995
DH2 DC1 DN1	107.735	157.600	DH9 DC6 DN2	113.656	210.032	DC16 DO3 DH25	109.653	63.407
DC1 DC3 DC4	114.293	301.432	DH10 DC6 DN2	107.550	148.929	DH24 DC16 DC17	109.121	255.136
DC1 DN1 DH1	116.690	286.102	DC6 DN2 DC13	123.918	191.909	DO3 DC16 DC17	108.786	243.184
DC1 DN1 C	122.115	342.629	DN2 DC13 DC14	137.173	295.808	DC16 DC17 DC18	112.675	350.196
DC1 DC3 DH3	106.035	193.662	DN2 DC13 DN6	119.945	230.345	DC16 DC17 DH26	107.886	220.937
DC1 DC3 DH4	109.300	242.980	DC13 DH20 DH20	119.864	254.587	DC16 DC17 DO4	110.373	267.441
DH1 DN1 C	120.922	284.339	DC13 DN6 DC12	108.415	340.424	DH26 DC17 DO4	109.649	190.630
DN1 C O	122.615	343.088	DC13 DC14 DH21	108.222	131.600	DC17 DO4 DH27	107.781	327.939
DN1 C CT	115.874	260.450	DC13 DC14 DN5	104.201	170.534	DO4 DC17 DC18	109.040	465.139
DN1 DC1 DC3	112.195	302.319	DC13 DC14 DC15	116.453	310.366	DH26 DC17 DC18	107.132	102.249
DC3 DC4 DC5	113.189	276.999	DH20 DN6 DC12	124.946	143.550	DC17 DC18 DO5	110.054	353.967
DC3 DC4 DH5	109.476	246.213	DN6 DC12 DN4	119.648	277.965	DC17 DC18 DH28	108.342	309.620
DC3 DC4 DH6	109.031	229.232	DN6 DC12 DN5	115.001	375.591	DC17 DC18 DH29	108.900	112.051
DH3 DC3 DH4	106.272	129.587	DN6 DC13 DC14	102.879	208.503	DC18 DO5 DH30	107.935	311.067

Triplet	Angle	FC	Triplet	Angle	FC	Triplet	Angle	FC
DH28 DC18 DO5	110.808	211.598	DH29 DC18 DO5	110.793	158.765	DH28 DC18 DH29	107.869	134.252
GH9 DN4 DC11	114.348	107.553	DN4 DC11 DH17	107.380	175.121	DN4 DC11 DH18	107.679	184.759
DH18 DC11 DH17	107.678	148.782	DH17 DC11 DC10	110.599	130.864	DH18 DC11 DC10	111.410	158.172
DC11 DC10 DH15	109.305	183.396	DC11 DC10 DH16	107.539	149.840	DH16 DC10 DH15	106.711	170.808
DH16 DC10 DC9	108.656	159.920	DH15 DC10 DC9	109.888	167.767	DN4 DC11 DC10	111.897	163.428
DC11 DC10 DC9	114.435	224.927	DC10 DC9 DC7	111.316	202.480	DH14 DC9 DH13	107.299	104.133
DH14 DC9 DC7	106.440	204.643	DH13 DC9 DC7	110.923	215.863	DC9 DC7 DH12	107.108	167.122
DC9 DC7 DN3	114.935	225.263	DC9 DC7 DC8	111.747	257.986	DH12 DC7 DC8	103.515	214.182
DH12 DC7 DN3	104.076	99.142	DC7 DN3 DH11	114.717	261.341	DC7 DN3 C	126.671	292.174
DH11 DN3 C	118.495	218.896	DN3 DC7 DC8	114.192	252.515	DN3 C O	123.580	258.603
DN3 C CT	115.812	240.349	DC7 DC8 DO2	119.489	382.461	DC7 DC8 N	117.706	414.026
DO2 DC8 N	122.773	429.218	DC8 N H	118.400	138.730	DC8 N CT	121.486	243.336

SI Table 3: The complete set of restrained atomic partial charge values for GODIC along with atomic pair bond length force constants (FC) in kJ mol^{-1} and corresponding equilibrium length (nm).

Atom	Charge	Atom	Charge	Bond	Length	FC	Bond	Length	FC
GN5	-0.3213	GH17	0.0681	GN1 GC12	0.125	175064.454	GC8 GC9	0.153	92665.366
GC1	-0.1032	GH18	0.0681	N GC13	0.134	194129.527	GC9 GN2	0.146	118069.249
GC13	0.6348	GN2	-0.7016	GC13 GO1	0.121	247488.448	GC7 GH13	0.108	38897.498
GO1	-0.5200	GH19	0.3772	GC13 GC1	0.153	61750.248	GC7 GH14	0.108	68978.337
GC2	-0.0926	GC10	0.7842	GC1 GH1	0.108	159269.846	GC8 GH15	0.109	51576.169
GC3	0.1286	GN4	-0.7900	GC1 GN5	0.145	116409.974	GC8 GH16	0.108	136736.918
GC4	-0.0002	GN3	-0.7167	GN5 GH2	0.100	201207.446	GC9 GH17	0.108	145990.235
GC5	0.0345	GH12	0.1095	GN5 C	0.136	167023.302	GC9 GH18	0.108	63132.810
GN1	-0.7034	GH11	0.0849	GC1 GC2	0.154	75726.923	GN2 GH19	0.100	213965.938
GH1	0.1061	GC12	0.7003	GC2 GC3	0.154	77503.248	GN2 GC10	0.135	197524.876
GH3	0.0331	GH20	0.3904	GC3 GC4	0.153	161472.264	GC10 GN3	0.127	235906.095
GH4	0.0331	GC11	0.1806	GC4 GC5	0.154	75284.156	GN3 GC11	0.145	57828.550
GH5	0.0006	GH21	0.0278	GC5 GN1	0.145	113535.046	GC11 GH21	0.108	108666.972
GH6	0.0006	GH22	0.0278	GC2 GH3	0.109	86270.049	GC11 GH22	0.109	91544.830
GH7	-0.0158			GC2 GH4	0.109	81149.230	GC11 GC12	0.153	96269.172
GH8	-0.0158			GC3 GH5	0.109	77043.771	GC12 GN4	0.139	70013.781

GH9	0.0581			GC3 GH6	0.108	31021.950	GN4 GC10	0.139	79721.197
GH10	0.0581			GC4 GH7	0.109	133280.148	GN4 GH20	0.100	176308.842
GH2	0.2315			GC4 GH8	0.109	43397.133			
GN6	-0.3220			GC5 GH9	0.109	38652.885			
GC6	-0.0110			GC5 GH10	0.108	122297.001			
GC14	0.5596			N GC14	0.135	65385.407			
GO2	-0.5605			GC14 GO2	0.120	67439.558			
GC7	0.0130			GC14 GC6	0.153	76638.497			
GH13	0.0379			GC6 GH11	0.108	122219.981			
GH14	0.0379			GC6 GN6	0.145	110350.523			
GC8	0.0119			GN6 GH12	0.100	75133.961			
GH15	0.0095			GN6 C	0.136	157482.871			
GH16	0.0095			GC6 GC7	0.154	95425.662			
GC9	0.0568			GC7 GC8	0.153	96859.655			

SI Table 4: The complete set of bond angle force constants (FC) in kJ mol^{-1} and corresponding equilibrium angle (degree) for GODIC.

Triplet	Angle	FC	Triplet	Angle	FC	Triplet	Angle	FC
GC7 GC8 GH16	109.899	117.540	GC2 GC3 GH5	109.188	104.360	GC6 GN6 GH12	116.463	119.374
GC8 GC9 GH17	109.563	89.247	GC2 GC3 GH6	109.878	330.892	GH12 GN6 C	117.191	137.689
GC8 GC9 GH18	110.927	235.972	GH5 GC3 GH6	106.348	135.044	GN6 C O	123.193	163.975
GC2 GC3 GC4	111.928	253.721	GH5 GC3 GC4	110.316	207.938	GN6 C CT	114.781	257.027
GC7 GC8 GH15	109.024	123.381	GH6 GC3 GC4	109.027	217.524	GC6 GC7 GC8	113.992	195.194
GC7 GC8 GH17	109.563	89.247	GC3 GC4 GC5	114.479	261.649	GC7 GC8 GC9	113.417	188.787
GC7 GC8 GH18	110.927	235.972	GC3 GC4 GH7	108.125	176.361	GC8 GC9 GN2	112.629	194.581
GN4 GC10 GN3	115.106	448.074	GC3 GC4 GH8	110.269	215.465	GC9 GN2 GH19	116.509	82.613
CT N GC13	121.758	309.827	GC4 GC5 GN1	114.063	197.876	GC9 GN2 GC10	122.052	255.634
H N GC13	117.909	146.613	GH7 GC4 GH8	106.034	156.650	GH19 GN2 GC10	116.419	105.958
N GC13 GO1	122.832	283.822	GH7 GC4 GC5	109.859	111.549	GH13 GC7 GH14	106.655	216.748
N GC13 GC1	117.087	424.555	GH8 GC4 GC5	107.750	227.968	GH15 GC8 GH16	106.848	203.998
GO1 GC13 GC1	119.915	338.460	GC4 GC5 GH9	108.611	176.826	GH17 GC9 GH18	107.470	92.838
GC13 GC1 GH1	105.620	208.972	GC4 GC5 GH10	108.607	234.225	GN6 GC6 GC7	111.969	301.470
GC13 GC1 GC2	109.621	509.974	GH9 GC5 GH10	106.440	154.355	GC6 GC7 GH13	110.006	215.890
GC13 GC1 GN5	111.971	256.997	GH9 GC5 GN1	112.184	275.038	GC6 GC7 GH14	106.700	244.832
GC1 GC2 GC3	113.614	195.721	GH10 GC5 GN1	106.592	177.239	GH13 GC7 GC8	109.946	164.854
GC1 GN5 GH2	117.216	103.950	GC5 GN1 GC12	120.448	249.020	GH14 GC7 GC8	109.236	166.534
GC1 GN5 C	120.147	269.623	GN1 GC12 GN4	122.378	323.895	GH15 GC8 GC9	109.092	223.252
GH1 GC1 GC2	110.078	244.702	GN1 GC12 GC11	134.522	196.278	GH16 GC8 GC9	108.350	171.429
GH1 GC1 GN5	109.690	167.903	CT N GC14	121.349	394.284	GH17 GC9 GN2	109.109	93.277
GN5 C O	121.597	229.835	H N GC14	118.086	220.946	GH18 GC9 GN2	106.978	226.927
GN5 C CT	115.731	189.496	N GC14 GO2	122.285	341.732	GN2 GC10 GN3	126.514	450.654
GH2 GN5 C	117.581	121.311	N GC14 GC6	116.401	273.639	GN2 GC10 GN4	118.374	418.047
GC1 GC2 GH3	108.616	264.451	GO2 GC14 GC6	121.287	241.842	GC10 GN4 GH20	123.890	119.718
GC1 GC2 GH4	108.507	313.210	GC14 GC6 GH11	105.376	259.155	GC10 GN3 GC11	106.925	280.179
GN5 GC1 GC2	109.786	271.144	GC14 GC6 GC7	111.568	266.137	GN3 GC11 GH21	111.254	148.418
GH3 GC2 GH4	106.380	126.597	GC14 GC6 GN6	112.339	249.443	GN3 GC11 GH22	111.286	242.859
GH3 GC2 GC3	110.466	173.209	GH11 GC6 GC7	109.180	163.296	GN3 GC11 GC12	105.275	201.552
GH4 GC2 GC3	108.989	243.480	GH11 GC6 GN6	105.991	172.857	GH21 GC11 GC12	111.633	210.387
GC3 GC4 GC5	114.479	261.649	GC6 GN6 C	122.001	191.635	GH22 GC11 GC12	110.125	175.959
GC11 GC12 GN4	103.060	198.902	GC12 GN4 GH20	119.855	198.164	GH22 GC11 GH21	107.322	158.064
GC10 GN4 GC12	107.818	322.893						

SI Table 5: The complete set of restrained atomic partial charge values for MODIC along with atomic pair bond length force constants (FC) in kJ mol^{-1} and corresponding equilibrium lengths (nm).

Atom	Charge	Atom	Charge	Bond	Length	FC	Bond	Length	FC
N	-0.3640	MH17	0.0877	N MC15	0.134	174753.594	MN3 MH11	0.099	220040.079
MC1	0.0358	MH18	0.0877	N MC14	0.134	135369.666	MC6 MN3	0.139	132751.924
MC15	0.4871	MN5	-0.7115	N MH25	0.099	223253.486	MC9 MN5	0.136	162164.312
MO1	-0.4941	MH16	0.3901	N MC1	0.146	100384.750	MN5 MH16	0.100	216598.544
MC2	-0.0624	MC9	0.7048	MC1 MH1	0.108	148053.942	MN5 MC10	0.146	132115.147
MC3	-0.0533	MN3	-0.6390	MC1 MC15	0.154	112759.394	MC10 MH17	0.108	86691.343
MC4	-0.0143	MN4	-0.6346	MC15 MO1	0.121	56253.436	MC10 MH18	0.108	99206.155
MC5	0.1407	MH24	0.2379	MC1 MC2	0.154	72652.266	MC10 MC11	0.153	99765.718
MN2	-0.6695	MH23	0.0963	MC2 MH3	0.109	60956.882	MC11 MH19	0.109	74234.069
MH1	0.0536	MC6	0.5494	MC2 MH4	0.109	102821.927	MC11 MH20	0.108	112883.873
MH3	0.0402	MH11	0.3547	MC2 MC3	0.154	85688.754	MC11 MC12	0.153	89555.723
MH4	0.0402	MC7	0.1213	MC3 MH5	0.108	100922.084	MC12 MH21	0.108	107746.374
MH5	0.0247	MC8	-0.0638	MC3 MH6	0.109	124592.394	MC12 MH22	0.109	87456.276
MH6	0.0247	MH13	0.0326	MC3 MC4	0.154	71186.388	MC12 MC13	0.154	100562.970
MH7	0.0209	MH14	0.0326	MC4 MH7	0.109	124616.450	MC13 MH23	0.108	118492.339
MH8	0.0209	MH15	0.0326	MC4 MH8	0.109	50616.955	MC13 MC14	0.153	103923.338
MH9	0.0315	MH12	0.0771	MC4 MC5	0.154	116670.315	MC14 MO2	0.121	277366.355
MH10	0.0315			MC5 MH9	0.108	153405.857	MC13 N	0.144	126114.268
MH25	0.2328			MC5 MH10	0.109	92826.373	N MH24	0.100	101831.908
N	-0.3498			MC5 MN2	0.145	111954.908			
MC13	-0.0761			MN2 MC6	0.125	310112.959			
MC14	0.5928			MC6 MC7	0.154	81432.229			
MO2	-0.5402			MC7 MH12	0.109	48536.776			
MC12	-0.0722			MC7 MC8	0.153	111904.813			
MH21	0.0501			MC8 MH15	0.108	152692.734			
MH22	0.0501			MC8 MH14	0.109	95472.241			
MC11	-0.0374			MC8 MH13	0.108	55702.491			
MH20	0.0417			MC7 MN4	0.146	86531.610			
MH19	0.0417			MN4 MC9	0.127	211807.726			
MC10	0.0164			MC9 MN3	0.138	133392.822			

Triplet	Angle	FC	Triplet	Angle	FC	Triplet	Angle	FC
N MC13 MC14	107.377	248.929	MH4 MC2 MH3	106.045	144.499	MH12 MC7 MC8	108.640	222.272
MN3 MC9 MN4	114.903	308.536	MH4 MC2 MC3	108.465	181.922	MC7 MC8 MH15	113.077	146.899
MC9 MN3 MH11	126.405	179.069	MH3 MC2 MC3	106.818	205.081	MC7 MC8 MH14	111.014	236.653
MN3 MC6 MC7	102.942	210.510	MC2 MC3 MH5	109.591	277.100	MC7 MC8 MH13	107.584	183.801
MH13 MC8 MH14	107.948	231.891	MC2 MC3 MH6	104.887	228.577	MN4 MC7 MC8	109.818	222.666
MH13 MC8 MH15	108.269	91.552	MC2 MC3 MC4	116.903	291.658	MC7 MN4 MC9	107.709	266.544
MH14 MC8 MH15	108.785	83.115	MH6 MC3 MH5	106.483	124.754	MN3 MC9 MN5	119.406	284.045
N MC1 MC2	113.137	295.520	MH6 MC3 MC4	108.350	87.414	MN4 MC9 MN5	125.690	289.386
MC15 MC1 MC2	114.359	286.781	MH5 MC3 MC4	110.020	278.706	MC9 MN5 MH16	114.793	161.080
MC1 MC2 MC3	122.896	225.873	MC3 MC4 MC5	113.423	273.473	MC9 MN5 MC10	118.066	220.302
C N MH25	118.232	171.304	MC3 MC4 MH7	109.677	150.917	MH16 MN5 MC10	114.749	178.492
C N MC1	122.668	250.406	MC3 MC4 MH8	110.410	197.788	MN5 MC10 MC11	111.981	261.062
CT N MC15	121.375	357.290	MH8 MC4 MH7	105.037	230.184	MN5 MC10 MH17	107.836	132.618
H N MC15	117.872	279.900	MH8 MC4 MC5	109.335	195.356	MN5 MC10 MH18	107.248	198.818
N MC15 MO1	122.756	299.683	MH7 MC4 MC5	108.606	207.426	MH18 MC10 MH17	107.722	109.635
N MC15 MC1	114.061	209.699	MC4 MC5 MH9	109.422	223.938	MH17 MC10 MC11	110.923	289.306
CT N MC14	123.621	213.668	MC4 MC5 MH10	108.977	201.132	MH18 MC10 MC11	110.939	219.414
H N MC14	118.310	258.605	MC4 MC5 MN2	110.075	184.842	MC10 MC11 MC12	113.316	403.455
N MC14 MO2	123.649	465.936	MH10 MC5 MH9	105.960	121.164	MC10 MC11 MH19	109.221	259.128
N MC14 MC13	115.351	272.231	MH10 MC5 MN2	110.456	123.210	MC10 MC11 MH20	108.012	242.441
C N MH24	120.893	188.901	MH9 MC5 MN2	111.839	169.827	MH17 MC10 MC11	110.923	289.306
C N MC13	121.989	243.539	MC5 MN2 MC6	121.624	253.653	MH18 MC10 MC11	110.939	219.414
MH25 N MC1	119.019	162.941	MN2 MC6 MN3	121.015	325.688	MH20 MC11 MH19	106.943	121.497
N MC1 MH1	105.268	133.912	MN2 MC6 MC7	136.043	220.511	H19 MC11 MC12		
N MC1 MC15	111.061	239.356	MC6 MN3 MH11	121.702	182.249	MH20 MC11 MC12	109.466	222.671
MO1 MC15 MC1	123.178	276.260	MC6 MN3 MC9	108.831	310.791	MC11 MC12 MC13	113.782	167.693
MH1 MC1 MC15	106.361	97.317	MC6 MC7 MH12	107.319	202.169	MC11 MC12 MH21	111.039	282.903
MH1 MC1 MC2	105.843	144.534	MC6 MC7 MN4	104.461	157.310	MC11 MC12 MH22	109.289	188.088
MC1 MC2 MH3	105.664	259.432	MC6 MC7 MC8	116.942	189.960	MH22 MC12 MC13	109.010	247.891
MC1 MC2 MH4	105.853	139.747	MH12 MC7 MN4	109.439	207.613	MH22 MC12 MH21	106.727	93.137

SI Table 6: The complete set of bond angle force constants (FC) in kJ mol^{-1} and corresponding equilibrium angle (degree) for MODIC.

Triplet	Angle	FC	Triplet	Angle	FC	Triplet	Angle	FC
MH21 MC12 MC13	106.726	167.571	MC12 MC13 MH23	107.934	211.149	MC12 MC13 MC14	111.358	253.871
MC12 MC13 N	112.523	229.703	MC13 MC14 MO2	120.989	281.304	MC13 N MH24	116.193	244.587
MH23 MC13 MC14	109.344	129.069	MH23 MC13 N	108.232	216.830			

SI Table 7: The equilibrium bond length (nm) of *special bond* atom pairs between two residues that make up a single cross-link.

Atom	Atom	Equilibration distance
MN2	MC6	0.125
GN1	GC12	0.125
DN2	DC13	0.125
PC6	PN6	0.146

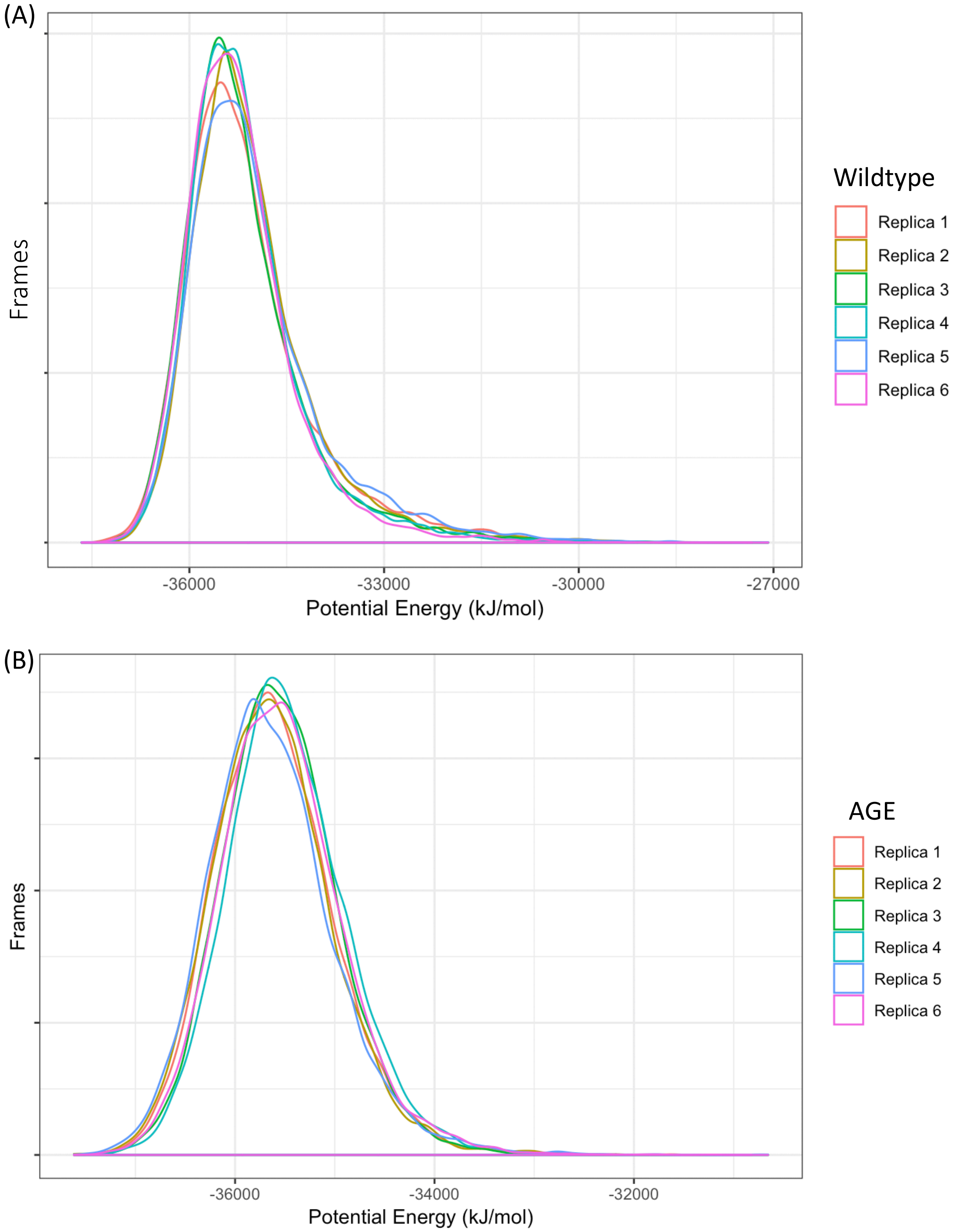

SI Figure 1: Distribution of potential energy over the production run for each replicate in the WT model (A) and AGE model (B).

(A)

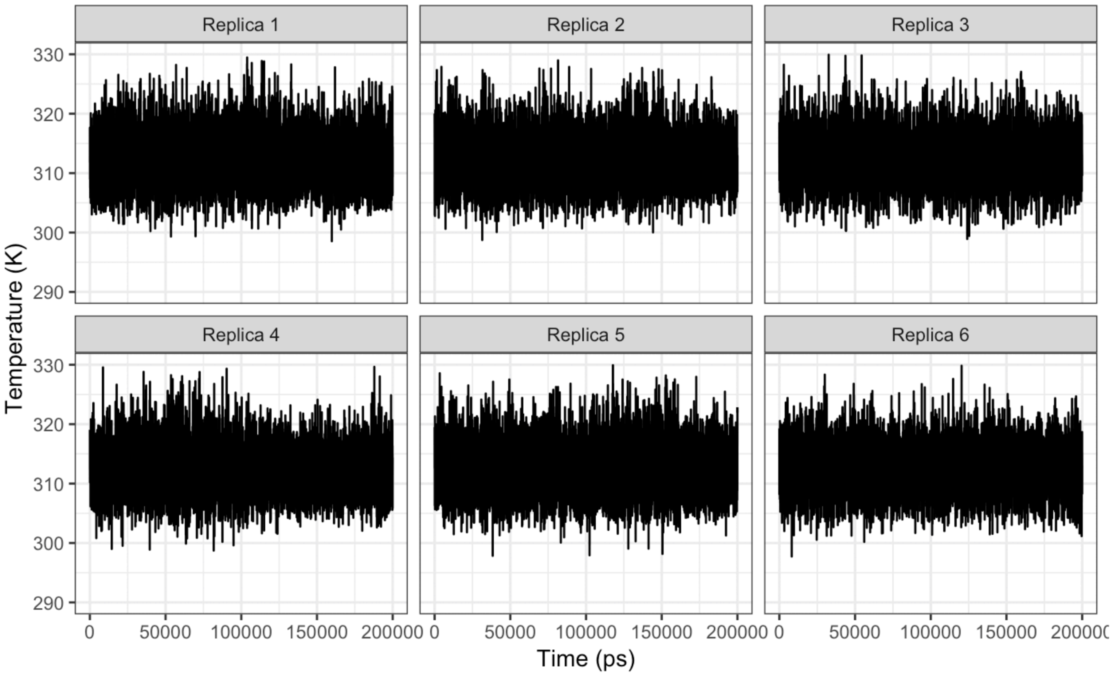

(B)

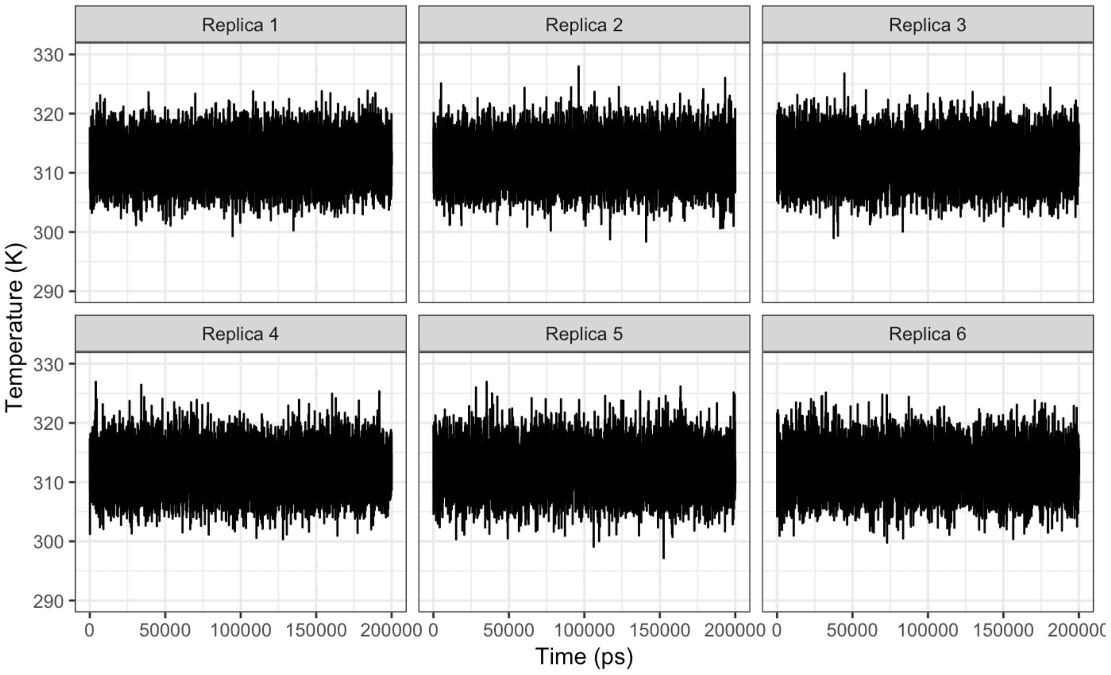

SI Figure 2: Time-series of temperature over the production run for each replicate in the WT model (A) and AGE model (B).

(A)

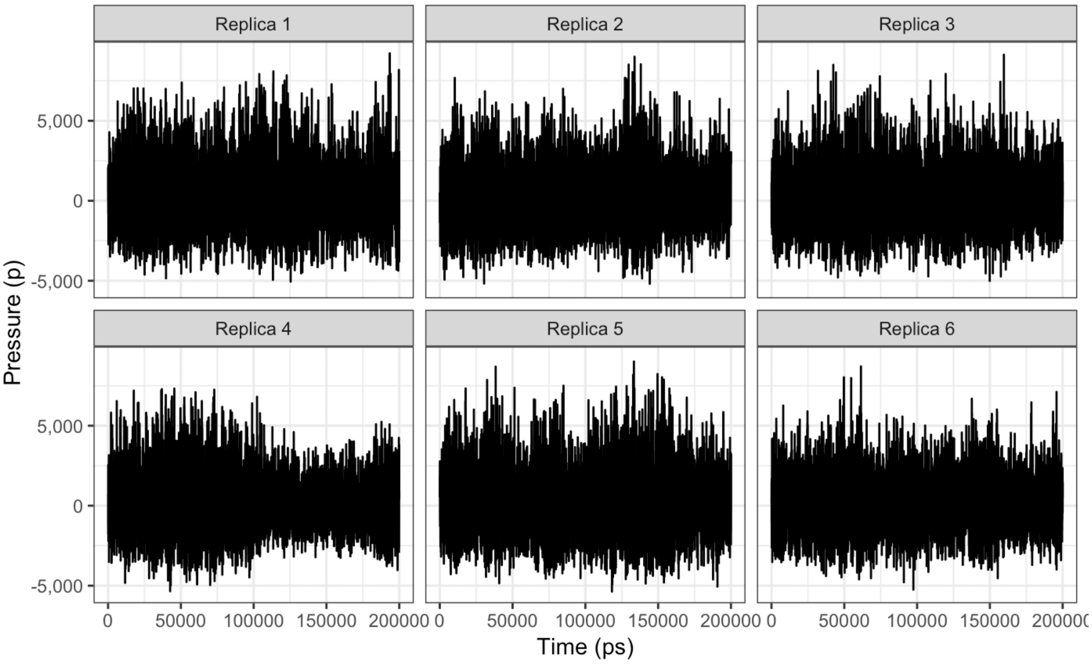

(B)

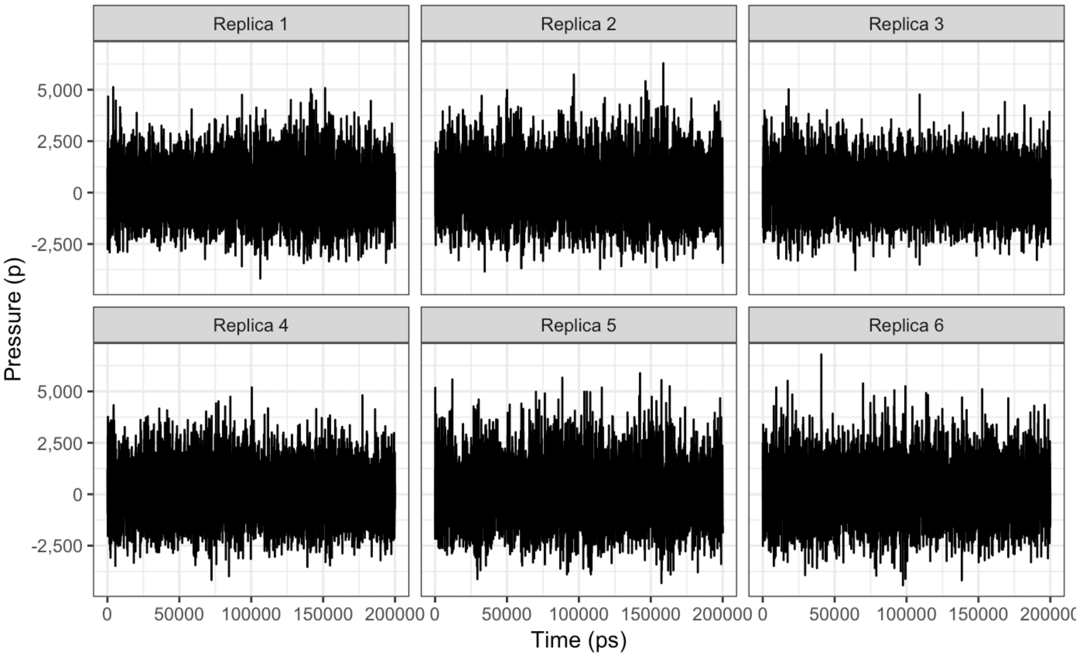

SI Figure 3: Time-series of pressure over the production run for each replicate in the WT model (A) and AGE model (B).

(A)

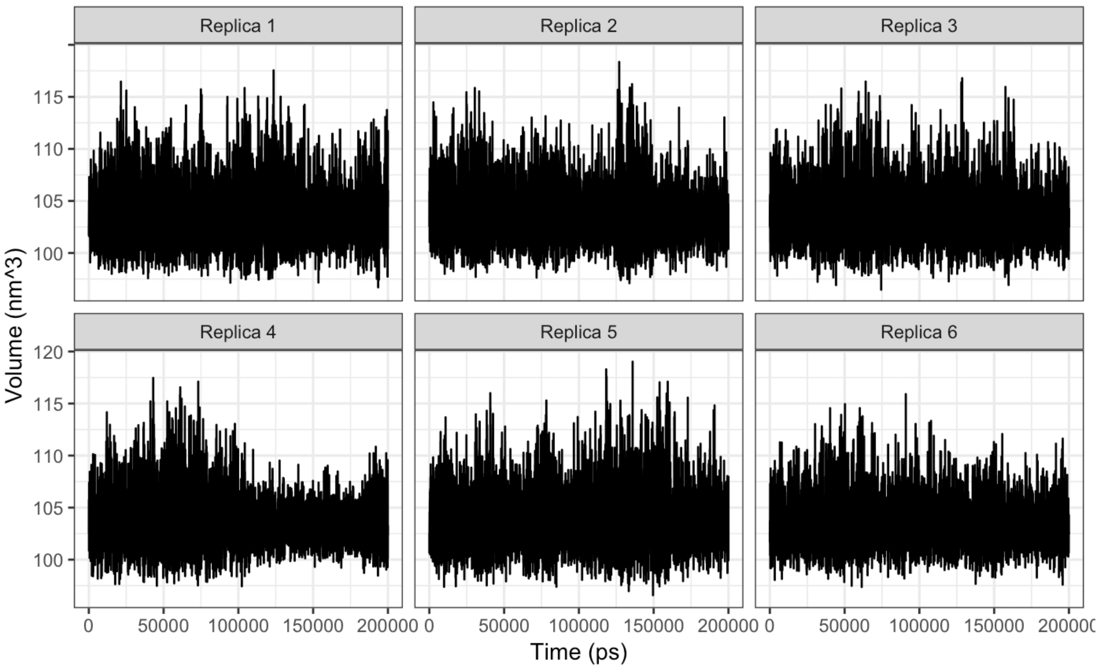

(B)

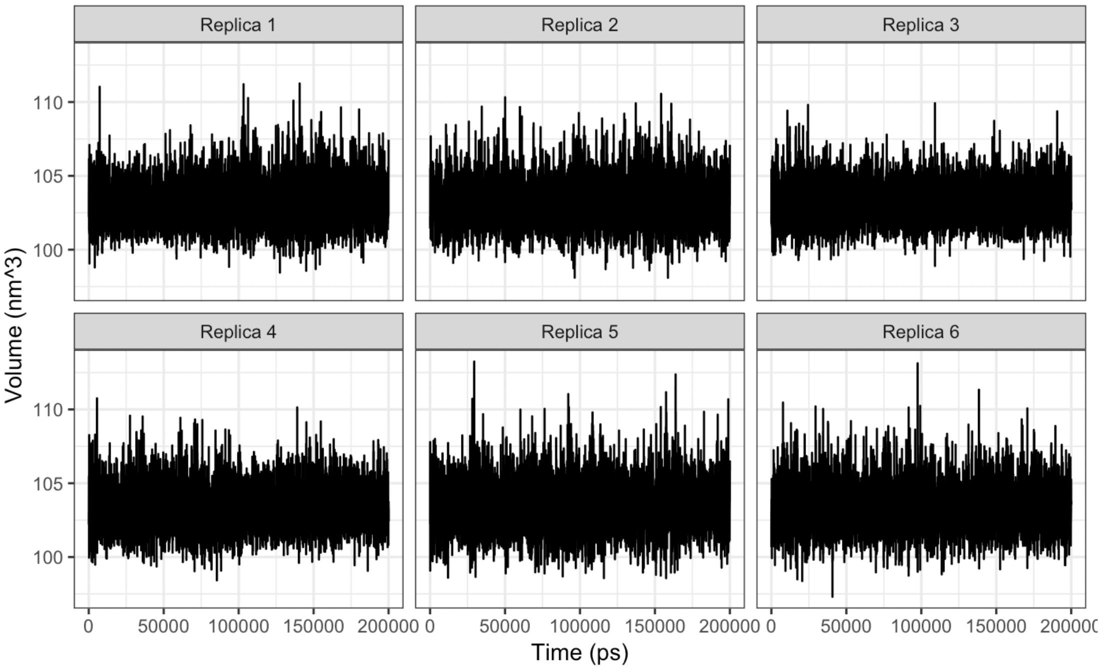

SI Figure 4: Time-series of unit cell volume over the production run for each replicate in the WT model (A) and AGE model (B).

(A)

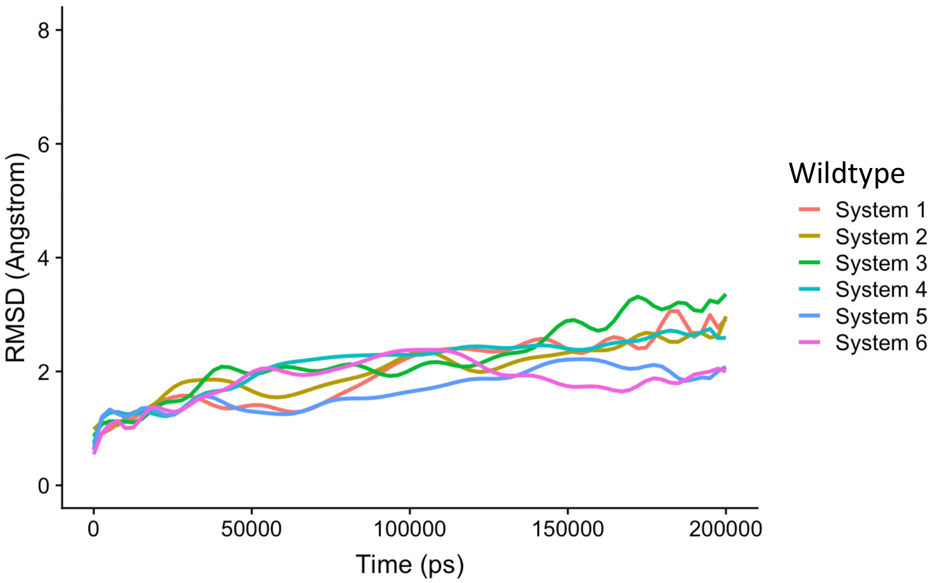

(B)

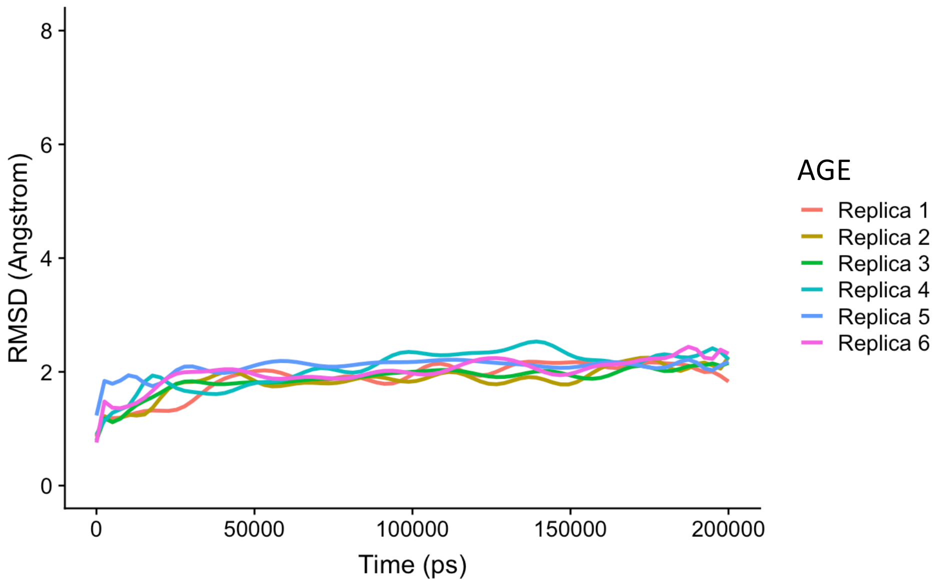

SI Figure 5: RMSD of collagen backbone heavy atoms of each replicate in the WT model (A) and AGE model (B).

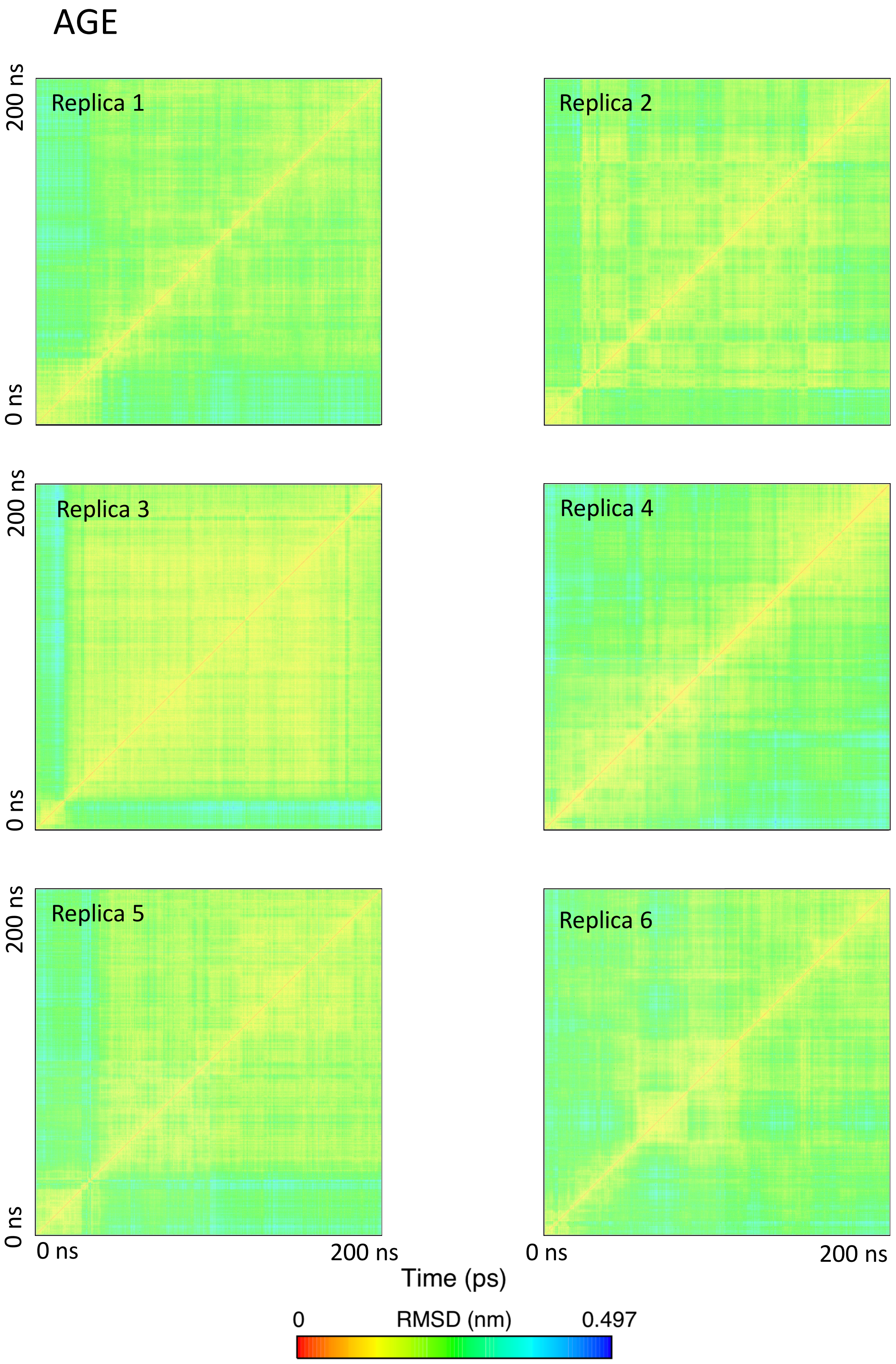

SI Figure 6: The RMSD of collagen backbone heavy atoms calculated across every time frame in the AGE model replicates.

Wildtyp

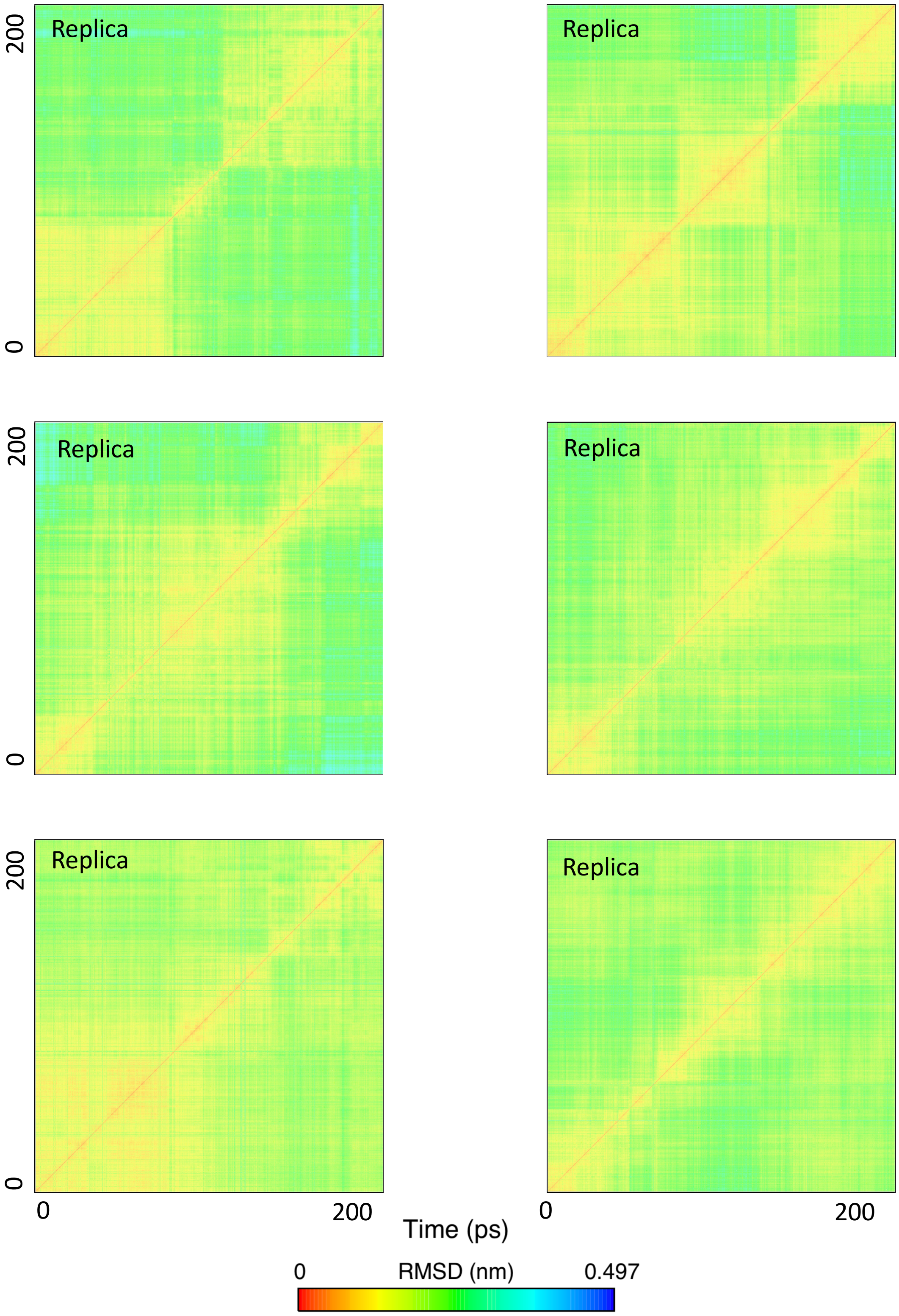

SI Figure 7: The RMSD of collagen backbone heavy atoms calculated across every time frame in the WT model replicates.

Wildtype

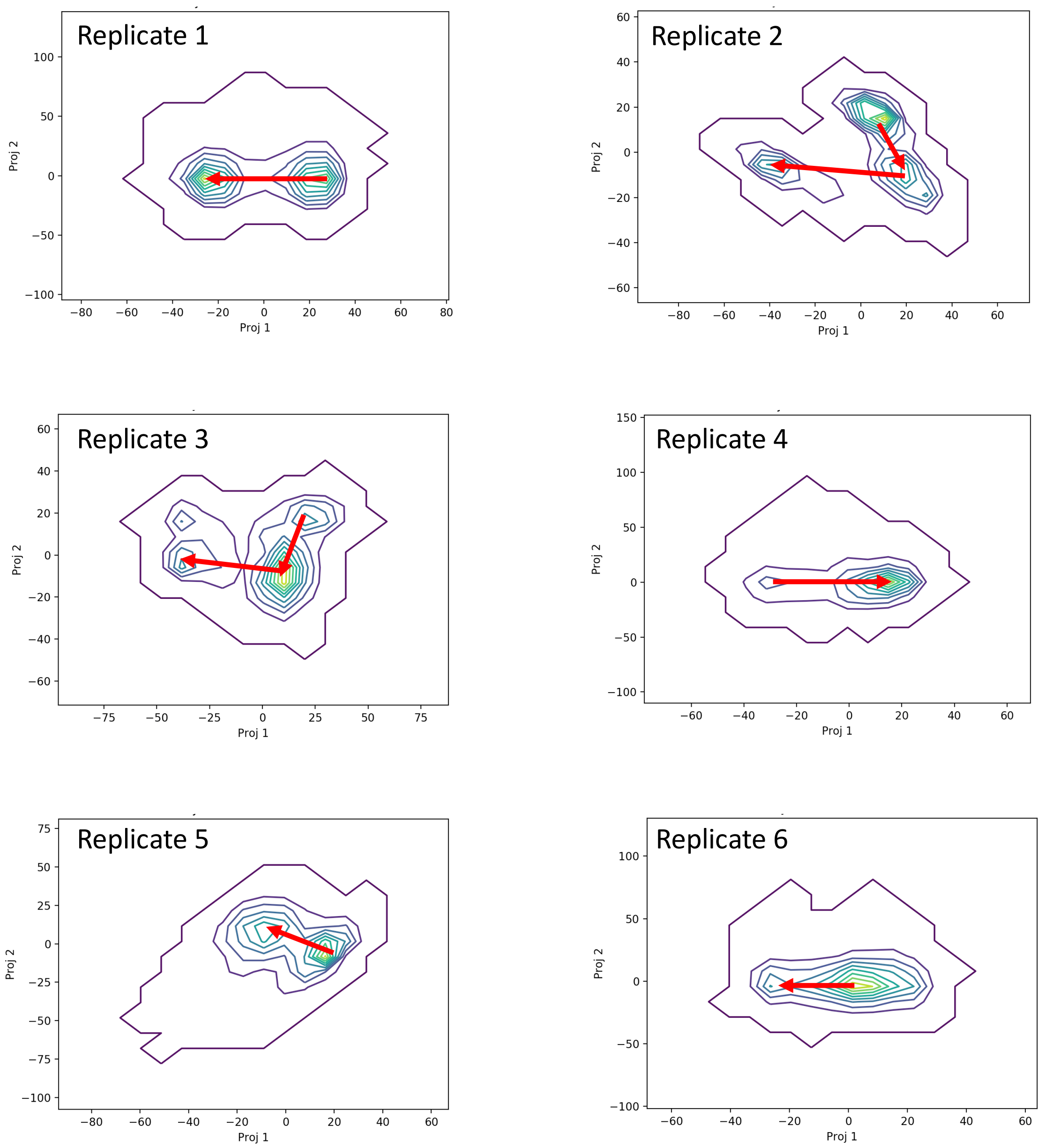

SI Figure 8: The projection of the WT model replicate simulations onto the conformational subspace defined by the PC1/PC2 for the backbone alpha-carbon. Arrows indicate the conformational sampling as time passes.

AGE

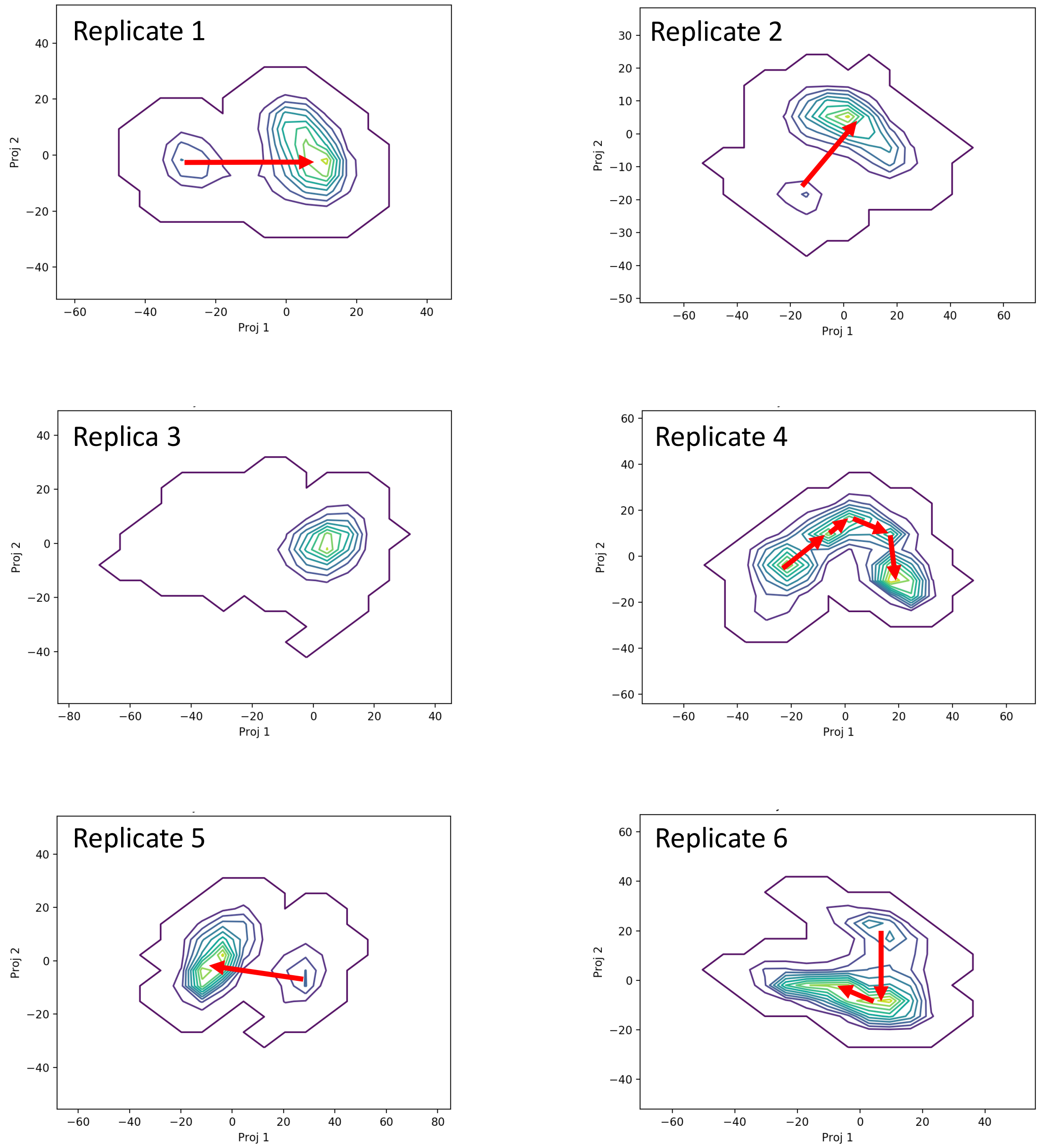

SI Figure 9: The projection of the AGE model replicate simulations onto the conformational subspace defined by the PC1/PC2 for the backbone alpha-carbon. Arrows indicate the conformational sampling as time passes.
